# Supplementary figures and images for: The notable global heterogeneity in the distribution of COVID-19 cases and the association with pre-existing parasitic diseases
Source: PLoS Negl Trop Dis. 2022 Oct 10;16(10):e0010826. doi: 10.1371/journal.pntd.0010826 (PMC9584393; doi:10.1371/journal.pntd.0010826)

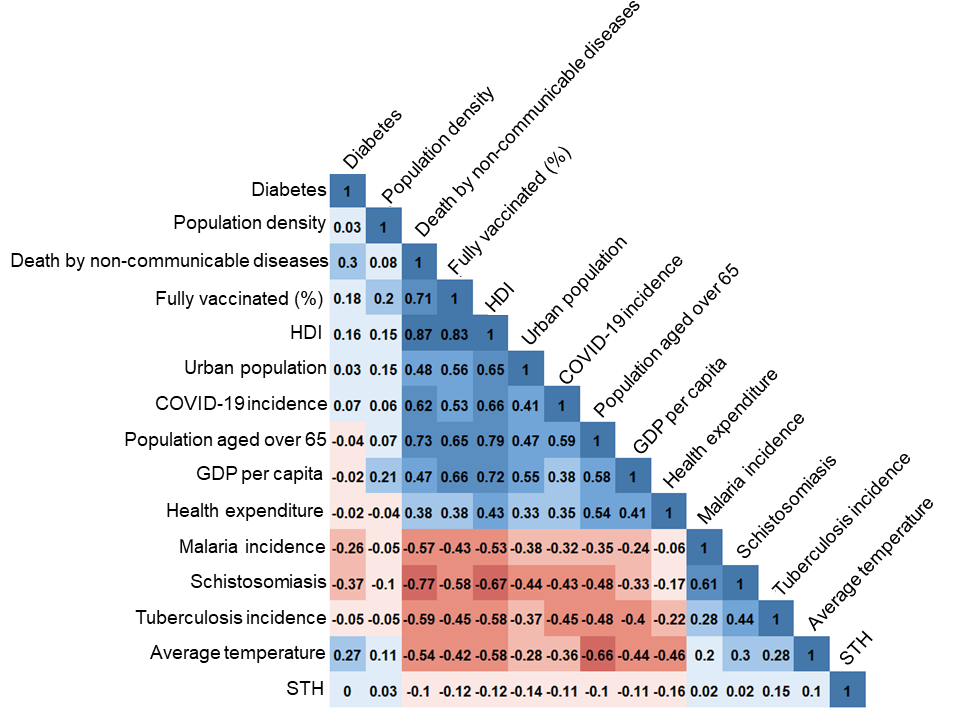

Supplement: S1 Fig — Values in each cell are the Pearson correlation coefficient for a given pair of variables. Coefficients close to -1 or +1 indicate high correlation, with 0 indicating minimal correlation. (TIF) [file pntd.0010826.s001.tif]
