# Supplementary material for: The notable global heterogeneity in the distribution of COVID-19 cases and the association with pre-existing parasitic diseases
Source: PLoS Negl Trop Dis. 2022 Oct 10;16(10):e0010826. doi: 10.1371/journal.pntd.0010826 (PMC9584393; doi:10.1371/journal.pntd.0010826)
Supplement: S1 Table — (PDF) [file pntd.0010826.s002.pdf]

Table S1. AIC and BIC of hierarchical models.

Model no.	Included variables	Adjusted ΔR^2	AIC	BIC
**1**	Case-incidence ~ **Parasite-variables**	0.24	483.83	440.30
**2**	Case-incidence ~ Parasite-variables + **Duration**	0.31	422.25	412.12
**3**	Case-incidence ~ Parasite-variables + Duration + **Population-over-65**	0.41	361.28	375.41
**4**	Case-incidence ~ Parasite-variables + Duration + Population-over-65 + **GDP**	0.49	324.65	357.55
