# Supplementary material for: The notable global heterogeneity in the distribution of COVID-19 cases and the association with pre-existing parasitic diseases
Source: PLoS Negl Trop Dis. 2022 Oct 10;16(10):e0010826. doi: 10.1371/journal.pntd.0010826 (PMC9584393; doi:10.1371/journal.pntd.0010826)
Supplement: Attachment — Submitted filename: PNTD-D-22-00241_answerofreviewer.pdf [file pntd.0010826.s005.pdf]

PLOS Neglected Tropical Diseases

The notable global heterogeneity in the distribution of COVID-19 cases and the association with pre-existing parasitic diseases

--Manuscript Draft--

**Manuscript Number:**	PNTD-D-22-00241R1
**Full Title:**	The notable global heterogeneity in the distribution of COVID-19 cases and the association with pre-existing parasitic diseases
**Short Title:**	Global pattern of COVID-19 and the association with parasitic diseases
**Article Type:**	Research Article
**Keywords:**	global disparity in the distribution of COVID-19; parasitic infections; malaria prevalence; country-level ecological study
**Abstract:**	Background<br>The coronavirus Disease 2019 (COVID-19) is a respiratory disease that has caused extensive ravages worldwide since being declared a pandemic by the World Health Organization (WHO). Unlike initially predicted by WHO, the incidence and severity of COVID-19 appeared milder in many Low-to-Middle-Income Countries (LMIC). To explain this noticeable disparity between countries, many hypotheses, including socio-demographic and geographic factors, have been put forward. This study aimed to estimate the possible association of parasitic diseases with COVID-19 as either protective agents or potential risk factors.<br><br>Methods/Principal findings<br>A country-level ecological study using publicly available data of countries was conducted. We conceptualized the true number of COVID-19 infections based on a function of test positivity rate (TPR) and employed linear regression analysis to assess the association between the outcome and parasitic diseases. We considered demographic, socioeconomic, and geographic confounders previously suggested. A notable heterogeneity was observed across WHO regions. The countries in Africa (AFRO) showed the lowest rates of COVID-19 incidence, and the countries in the Americas (AMRO) presented the highest. The multivariable model results were computed using 165 countries, excluding missing values. In the models analyzed, lower COVID-19 incidence rates were consistently observed in malaria-endemic countries, even accounting for potential confounding variables, Gross Domestic Product (GDP) per capita, the population aged 65 and above, and differences in the duration of COVID-19. However, the other parasitic diseases were not significantly associated with the spread of the pandemic.<br><br>Conclusions/Significance<br>This study suggests that malaria prevalence is an essential factor that explains variability in the observed incidence of COVID-19 cases at the national level. Potential associations of COVID-19 with schistosomiasis and soil-transmitted helminthiases (STHs) are worthy of further investigation but appeared unlikely, based on this analysis, to be critical factors of the variability in COVID-19 epidemic trends. The quality of publicly accessible data and its ecological design constrained our research, with fundamental disparities in monitoring and testing capabilities between countries. Research at the subnational or individual level should be conducted to explore hypotheses further.
**Additional Information:**	
**Question**	**Response**
**Financial Disclosure**<br><br>Enter a financial disclosure statement that describes the sources of funding for the work included in this submission. Review the submission guidelines for detailed	The author(s) received no specific funding for this work.

requirements. View published research articles from *PLOS NTDs* for specific examples.<br><br>This statement is required for submission and **will appear in the published article** if the submission is accepted. Please make sure it is accurate.<br><br>**Unfunded studies**<br><br>Enter: *The author(s) received no specific funding for this work.*<br><br>**Funded studies**<br><br>Enter a statement with the following details:<br>• Initials of the authors who received each award<br>• Grant numbers awarded to each author<br>• The full name of each funder<br>• URL of each funder website<br>• Did the sponsors or funders play any role in the study design, data collection and analysis, decision to publish, or preparation of the manuscript?<br>• **NO** - Include this sentence at the end of your statement: *The funders had no role in study design, data collection and analysis, decision to publish, or preparation of the manuscript.*<br>• **YES** - Specify the role(s) played.<br><br>* typeset	
**Competing Interests**<br><br>Use the instructions below to enter a competing interest statement for this submission. On behalf of all authors, disclose any competing interests that could be perceived to bias this work—acknowledging all financial support and any other relevant financial or non-	The authors have declared that no competing interests exist.

financial competing interests.<br><br>This statement **will appear in the published article** if the submission is accepted. Please make sure it is accurate. View published research articles from *PLOS NTDs* for specific examples.<br><br>**NO authors have competing interests**<br><br>Enter: *The authors have declared that no competing interests exist.*<br><br>**Authors with competing interests**<br><br>Enter competing interest details beginning with this statement:<br><br>*I have read the journal's policy and the authors of this manuscript have the following competing interests: [insert competing interests here]*<br><br>* typeset<br><br>This statement is required for submission and **will appear in the published article** if the submission is accepted. Please make sure it is accurate and that any funding sources listed in your Funding Information later in the submission form are also declared in your Financial Disclosure statement.	
**Data Availability**	Yes - all data are fully available without restriction

Powered by Editorial Manager® and ProduXion Manager® from Aries Systems Corporation

Authors are required to make all data underlying the findings described fully available, without restriction, and from the time of publication. PLOS allows rare exceptions to address legal and ethical concerns. See the PLOS Data Policy and FAQ for detailed information.<br><br>A Data Availability Statement describing where the data can be found is required at submission. Your answers to this question constitute the Data Availability Statement and **will be published in the article**, if accepted.<br><br>**Important:** Stating 'data available on request from the author' is not sufficient. If your data are only available upon request, select 'No' for the first question and explain your exceptional situation in the text box.<br><br>Do the authors confirm that all data underlying the findings described in their manuscript are fully available without restriction?	
**Describe where the data may be found in full sentences. If you are copying our sample text, replace any instances of XXX with the appropriate details.**<br><br>• If the data are **held or will be held in a public repository**, include URLs, accession numbers or DOIs. If this information will only be available after acceptance, indicate this by ticking the box below. For example: *All XXX files are available from the XXX database (accession number(s) XXX, XXX.*).<br>• If the data are all contained **within the**	All epidemiological and socio-economic data are available from the Global Health Observatory (https://www.who.int/data/gho/data/indicators) and World Development Indicators (https://databank.worldbank.org/source/world-development-indicators/Type/TABLE/preview/on#) database

Powered by Editorial Manager® and ProduXion Manager® from Aries Systems Corporation

**manuscript and/or Supporting Information files**, enter the following: *All relevant data are within the manuscript and its Supporting Information files.*<br>• If neither of these applies but you are able to provide **details of access elsewhere**, with or without limitations, please do so. For example:<br><br>*Data cannot be shared publicly because of [XXX]. Data are available from the XXX Institutional Data Access / Ethics Committee (contact via XXX) for researchers who meet the criteria for access to confidential data.*<br><br>*The data underlying the results presented in the study are available from (include the name of the third party and contact information or URL).*<br>• This text is appropriate if the data are owned by a third party and authors do not have permission to share the data.<br><br>* typeset	
Additional data availability information:	

Manuscript

Click here to access/download;Manuscript;COVID-19-parasite-manuscript_revision_sub.docx

RESEARCH ARTICLE

The notable global heterogeneity in the distribution of COVID-19 cases and the association with pre-existing parasitic diseases

Taehee Chang[1], Bong-Kwang Jung[2], Jong-Yil Chai[3], Sung-il Cho[1,4]* (Orcid; 0000-0003-4085-1494)

[1]Department of Public Health Sciences, Graduate School of Public Health, Seoul National University, Seoul, Republic of Korea

[2]Institute of Parasitic Diseases, Korea Association of Health Promotion, Seoul, Republic of Korea

[3]Department of Tropical Medicine and Parasitology, Seoul National University College of Medicine, Seoul, Republic of Korea

[4]Institute of Health and Environment, Seoul National University, Seoul, Republic of Korea

*Corresponding author: Sung-il Cho

E-mail: scho@snu.ac.kr (SC)

Abstract

Background

The coronavirus Disease 2019 (COVID-19) is a respiratory disease that has caused extensive ravages worldwide since being declared a pandemic by the World Health Organization (WHO). Unlike initially predicted by WHO, the incidence and severity of COVID-19 appeared milder in many Low-to-Middle-Income Countries (LMIC). To explain this noticeable disparity between countries, many hypotheses, including socio-demographic and geographic factors, have been put forward. This study aimed to estimate the possible association of parasitic diseases with COVID-19 as either protective agents or potential risk factors.

Methods/Principal findings

A country-level ecological study using publicly available data of countries was conducted. We conceptualized the true number of COVID-19 infections based on a function of test positivity rate (TPR) and employed linear regression analysis to assess the association between the outcome and parasitic diseases. We considered demographic, socioeconomic, and geographic confounders previously suggested. A notable heterogeneity was observed across WHO regions. The countries in Africa (AFRO) showed the lowest rates of COVID-19 incidence, and the countries in the Americas (AMRO) presented the highest. The multivariable model results were computed using 165 countries, excluding missing values. In the models analyzed, lower COVID-19 incidence rates were consistently observed in malaria-endemic countries, even accounting for potential confounding variables, Gross Domestic Product (GDP) per capita, the population aged 65 and above, and differences in the duration of COVID-19. However, the other parasitic diseases were not significantly associated with the spread of the pandemic.

Conclusions/Significance

This study suggests that malaria prevalence is an essential factor that explains variability in the observed incidence of COVID-19 cases at the national level. Potential associations of COVID-19 with schistosomiasis and soil-transmitted helminthiases (STHs) are worthy of further investigation but appeared unlikely, based on this analysis, to be critical factors of the variability in COVID-19 epidemic trends. The quality of publicly accessible data and its ecological design constrained our research, with fundamental disparities in monitoring and testing capabilities between countries. Research at the subnational or individual level should be conducted to explore hypotheses further.

Author summary

SARS-CoV-2 has spread fast worldwide, yet the first pandemic waves in LMICs looked weaker than mathematical models had predicted. Suggestions for this observed disparity include socio-demographic and geographic factors and immunological modulation caused by exposure to endemic parasite infections. In countries where parasitic diseases are widespread, particularly in Sub-Saharan Africa (SSA), residents may have been coinfected with COVID-19 and pre-existing parasitic diseases due to

the rapid spread of SARS-CoV-2. Therefore, we performed a nation-level ecological analysis to describe trends in primary SARS-CoV-2 outcomes by country and investigate potential correlations between these outcomes and pre-existing parasitic diseases. Our results suggest that malaria prevalence is a crucial factor to explain variation in COVID-19 incidence at the national scale, with a substantial relationship persisting even when putative confounders were adjusted. While we note that causal inference cannot be proved owing to insufficient data and hidden confounders, this will aid in generating new hypotheses and identifying intervention strategies to reduce NTDs and malaria in the context of the coronavirus pandemic.

Introduction

COVID-19, caused by the novel SARS-CoV-2 virus (Coronaviridae), has reached all corners of the world, becoming the worst epidemic in the century. Since the disease outbreak, researchers have insisted on a dramatic surge of COVID-19 in Low-to-Middle-Income Countries (LMICs) compared to developed countries with better infrastructures and health policies [1,2]. However, contrary to expectations, the pandemic has caused remarkably higher morbidity and mortality consequences in developed countries. Sub-Saharan Africa (SSA), a region already beset by a lack of health infrastructure, poverty, and the highest rates of Neglected Tropical Diseases (NTDs), seem relatively spared, while other regions of the world (such as the USA and Europe) have been bearing the tremendous burden of cases and associated deaths [3]. SSA is the least affected continent, making up 2.5 % of worldwide cases though accounting for 14 % of the worldwide population (Fig 1).

Fig 1. Incidence rates of COVID-19 by WHO regions and their trends. (A) The proportion of incidence rates of COVID-19 by WHO region; (B) Trends in the average number of confirmed cases per million people from March 2020 to October 2021. AMR - Americas; EUR - Europe; SEAR - Southeast Asia; EMR - East Mediterranean; WPR - Western Pacific; AFR - Africa.

Several hypotheses have been posited to explain why the transmission and severity of COVID-19 in LMICs remain comparatively low despite limited laboratory capacity, weak healthcare infrastructure, and a lack of public health system [2,4-10]. One argument raised is that the number of cases and deaths in the regions is underreported due to limited testing capacity, though the degree is uncertain [2]. Other theories have suggested that the presence of non-communicable diseases, including diabetes, cerebrovascular diseases, and hypertension, influence the severity of COVID-19 consequences [8,10].

In LMICs, the population structure with a smaller proportion of older people who are more susceptible to infection and death could have played an essential role in curbing the spread of the coronavirus [2,5,9]. On the other hand, it has been suggested that urbanization, dense population, or cold climate can enhance coronavirus transmission, resulting in a surge of COVID-19 in developed countries [2,11]. In addition to the above factors, previous studies documented several socioeconomic indicators, including Gross Domestic Product (GDP) per capita, Human Development Index (HDI), and differences in the timing of disease onset to explain noticeable geographical differences in the burden of COVID-19 [2,5].

NTDs, including schistosomiasis and soil-transmitted helminthiases (STHs), are primarily parasitic diseases that cause significant morbidity and mortality, mainly in LMICs [12]. They affect more than 2 billion people worldwide, with disproportionately high prevalence in tropical and subtropical countries' most deprived and poorest areas [13]. Moreover, malaria is also rampant in the regions already suffering from the burden of poverty and NTDs, with more than 200 million cases per year and 400,000 associated deaths in 2018 (> 90 % in SSA) [14]. With the rapid increase of COVID-19, inhabitants in the countries where the parasitic diseases are endemic, especially in SSA, have possibly been coinfected with COVID-19 and pre-existing parasitic diseases.

Human-infecting parasites have coevolved with their hosts during evolutionary processes, with persistent immune system modulation through molecular and physiological mechanisms [15-18]. Helminths or their excretory-secretory products (ES) are recognized by the host immune system, known as microorganism-associated molecular patterns (MAMPs) or pathogen-associated molecular patterns (PAMPs) [19]. Though it depends on at which stage the helminths are in their life cycle, generally, a modulated T-helper 2 (Th2) response is triggered, resulting in a hypo-responsive state, which is characterized by the stimulation of dendritic cells (DCs), eosinophils, basophils, mast cells, alternatively activated macrophages (AAMs), regulatory B cells (Bregs), and regulatory T-cells (Tregs) [17-20]. The systemic immunomodulatory effects caused by helminths are a "double-edged sword" as those can potentially support viral infection [21,22], affect the efficiency of vaccines [23], or protect the human host from detrimental consequences of COVID-19 by reducing inflammatory reactions [15,24,25].

Five species of the genus *Plasmodium* are known to cause malaria in humans (Table 1). Clinical symptoms of malaria occur with the rupture of infected erythrocytes, causing stimulation of immune response and resulting in the release of pro-inflammatory cytokines including IL-6, IL-12, TNF-alpha, and interferon-gamma [26]. Some patients infected with COVID-19 experience a systemic inflammatory response, called "cytokine storm," due to excessive activation of T-helper 17 (Th17) [27,28], which similarly appear in some severe manifestations of malaria induced by pro-inflammatory

T-helper 1 (Th1)-related immune response [26,29]. Therefore, the resemblance of immune reactions suggests that coinfection of COVID-19 and malaria might lead to synergistic epidemics and more severe manifestations [26].

Table 1. Summary of principal characteristics of malaria, schistosomiasis, and soil-transmitted helminths.

Characteristics	Malaria	Schistosomiasis	Soil-transmitted helminths
**Principal symptoms**	Fever, nausea, fatigue	Acute infection causes diarrhea, abdominal pain, cough, fever, and eosinophilia; Chronic infections are often asymptomatic	Abdominal pain, nausea, anemia and diarrhea; Chronic infections are often asymptomatic
**Infectious agent**	*Plasmodium vivax, P. falciparum, P. malariae, P. ovale,* and *P. knowlesi*	*Schistosoma haematobium, S. japonicum,* and *S. mansoni*	*Trichuris trichiura, Ascaris lumbricoides,* and hookworm (*Necator americanus* and *Ancylostoma duodenale*)

The COVID-19 outbreak has prompted researchers to conduct nationwide comparisons to assess the effect of the pandemic. However, there are particular difficulties with interpreting comparable COVID-19 data among countries with different testing capacities, political regimes, and reporting processes [30]. Models employing transmission simulations and flight data have been used to estimate the number of undetected incidents [31,32]. These methods often need location-specific inputs, which restricts their adaptability and scalability [33]. Alternatively, the infection-fatality rate (IFR) or case-fatality rate (CFR) of COVID-19 based on serological testing and extensive diagnostic testing has been utilized to determine the actual extent of the pandemic in previous cases [34,35]. However, the IFR can be impacted by variables, including the political system, demographics, and health system capability. Furthermore, the degree to which the mortality count is accurate varies depending on how COVID-19-related fatalities are defined and how testing is conducted [33]. Researchers should employ a straightforward, comprehensive measure that is scalable across all nations and regions to enable statistical methods like multiple linear regression so they may fully evaluate the real amount of morbidity brought on by COVID-19.

This study aimed to identify the potential influence of pre-existing parasitic infections on COVID-19 morbidity. The study also considered demographic, socioeconomic, and geographic variables documented in previous epidemiological studies. We conducted an ecological study using country-level data to describe patterns in COVID-19 incidence rates and explore possible associations of parasitic diseases with COVID-19 while adjusting the effect of confounding factors. Our results will be essential for developing prevention and control strategies to reduce NTDs and malaria in the context of the coronavirus pandemic.

Methods

Data extraction and descriptive analysis

We utilized publicly available data from each country affected by the coronavirus pandemic; observed COVID-19 confirmed cases from March 2020 to October 2021 were included [3]. The cumulative number of COVID-19 cases was counted and adjusted for the population size. The number of analysis units of the data is documented in Table 2.

Table 2. Summary statistics of the variables included in the study.

Variable	n	Year	Mean	Median	sd	Min	Max
Incidence of COVID-19 cases (in %)	202	2021	5.25	4.06	5.19	0.00	23.21
Malaria (estimated prevalence of Malaria based on confirmed cases, in %)	175	2017	2.58	0.00	7.77	0.00	64.50
Schistosomiasis (proportion of individuals requiring preventive chemotherapy, in %)	172	2019	4.78	0.00	10.17	0.00	50.31
STHs (proportion of children requiring preventive chemotherapy, in %)	179	2019	0.44	0.00	2.88	0.00	41.66
Tuberculosis (prevalence of new and relapse cases, in %)	187	2019	0.06	0.03	13.32	0.00	0.32
Cause of death, by non-communicable diseases (in %)	184	2019	70.41	77.48	20.54	26.99	96.13
Diabetes (in % of population ages 20 to 79)	202	2019	8.39	6.90	4.75	1.00	30.50
Population ages 65 and above (in %)	188	2020	9.23	7.01	6.53	1.26	28.39
Current health expenditure (% GDP)	187	2018	6.48	6.33	2.86	1.59	19.04

GDP per capita (in $1,000)	193	2018	16.49	6.61	24.78	0.27	114.68
Human development index	187	2018	0.72	0.74	0.15	0.39	0.97
Urban population (in %)	202	2020	61.18	62.11	22.34	13.35	100.00
Population density (per km²)	198	2019	318.59	82.33	648.73	0.14	7915.73
Average surface temperature (in ℃)	196	2017	20.20	23.77	8.51	-15.41	30.74
The number of days elapsed from the first case of COVID-19 in China until the first cases in each country (in days)	193	2021	76.64	69.00	77.69	3.00	668.00
Fully vaccinated with COVID-vaccine (in %)	199	2021	37.89	33.24	26.72	0.04	87.01

We collated the data on predictive indicators available from various sources, including Global Health Observatory (GHO) [36] and World Development Indicators (WDI) [37]. Information on the variables employed is detailed (Table 2). Of a diverse group of human infecting parasites, only *Schistosoma* spp., STHs, and *Plasmodium* spp. were considered in this study (Table 1). The parasitic disease variables were categorized to represent the state of endemicity based on the number of confirmed cases or individuals who require preventive chemotherapy.

The epidemiological factors included in the analysis as predictive variables consist of the prevalence of tuberculosis, the proportion of death caused by non-communicable diseases, and the prevalence of diabetes. Based on the WHO report [38], these variables were selected, suggesting that patients with pre-existing medical disorders are more susceptible to coronavirus infection. Though people of all ages can be infected, older people (e.g., older than 65) are at higher risk [39]. Therefore, we considered the proportion of the population aged 65 and over as a demographic factor to reflect the age structure. As for socioeconomic variables, we used current health expenditure to measure health infrastructure, GDP per capita to measure income, and the HDI to measure overall living standard [2]. Following related literature [2,5], we considered population density, the degree of urbanization (the proportion of people residing in urban areas), and mean surface temperature (℃) for geographic factors. We denoted several more elements, the variables that account for the timing of the disease occurrence (the number of days since the first incidence of COVID-19 in China until the first cases in each country), and the proportion of the population vaccinated with the COVID-19 vaccine.

Outcome adjustment

We evaluated the association between the disease outcome and indicators of parasitic diseases using a linear regression model framework. However, due to uncertainties in data quality, we were only able to examine the number of reported cases as an available proxy for the real COVID-19 outcome. Therefore, we conceptualized the true number of the infections (*Inf*) to be a function of factor *f* and reported cases *C* (Eq 1).

$$Inf = fC \tag{1}$$

To create a numerical value for *f*, two versions of *f* (f_1 and f_2) were considered following a previous study [33]; f_1 was obtained using CFR, while f_2 utilized TPR. The factor f_1 was suggested to effectively reflect *Inf* since CFR has been employed as a real-time way to monitor underreporting and an indication of the actual prevalence of an infectious illness in previous situations [34,35]. Nonetheless, mortality statistics may also be underreported in areas with insufficient or low testing levels, making CFR a less representative indication. Therefore, in this study, the simpler and more comprehensive factor f_2 was utilized to estimate *Inf* (Eq2), based on test positivity rate (TPR) - an indication of appropriate testing in relation to disease prevalence (Eq3). The data on the number of tests per million was collected from WDI [37].

$$f_2 = \begin{cases} 1 & , \quad if\ TPR < 0.05 \\ TPR/0.05 & , \quad if\ TPR \geq 0.05 \end{cases} \tag{2}$$

$$TPR = \frac{C}{T} \tag{3}$$

When TPR is less than 5%, the World Health Organization (WHO) recommends that testing capacity is acceptable [40]. If TPR was more than 5 percent, it was assumed that a growing number of instances were being unreported and that the factor f_2 would be equal to the TPR's ratio to that of 5 percent, the threshold (Eq2). The estimated *Inf* was used as a response variable in linear regression models in this study.

Statistical modeling

The β coefficients and 95 % confidence intervals (95 % CIs) were estimated based on the root-transformed COVID-19 incidence rates. The level of statistical significance was set at 0.05. All analysis was conducted on R software [41]. As the response variable was continuous, a least-squares fixed-effect linear regression model was applied to explore possible associations.

We considered the square root transformation of the outcome variable, *Inf*, since the variable was not normally distributed. Then, we investigated whether continuous predictive variables could be assumed to have linearity with root-transformed COVID-19 incidence rates by fitting a generalized additive model (GAM). Most predictors had a non-linear relationship with the response variable and showed better fitting once categorized than continuous forms. Therefore, to maintain consistency through the variables, all predictive variables were categorized into ordered quantiles or according to clusters that appeared on a scatterplot.

Classic standard errors (SE) may not be accurately estimated due to the small sample size and skewed outcome distribution. We, therefore, presented both traditional SE and 95 % CI, and robust SE and 95 % CI computed using the bootstrap method (boot package, R = 2,000) [42,43] as a complementary estimation approach.

Multivariable linear regression

We applied univariable and multivariable linear regression analysis to investigate the associations. All predictive variables were tested first in univariable regression, and only the variables significantly associated with the outcome were included in the multivariable model. To avoid the problem of multicollinearity, we first observed pair-wise Pearson correlation coefficients and scatterplots (S1 Fig). We screened variables that could cause multicollinearity, and within a group of correlated variables, the variables with fewer missing values and more substantial statistical power were selected. Subsequently, a stepwise forward selection procedure based on parasitic disease variables retained the number of predictors to the level at which the adjusted R^2 (R_a^2) is not higher above the R_a^2 of the complete model. The selection step was carried out by referring to the study that documented the importance of restraining alpha significance level and preventing overestimation of the explained variance [44s]. We fit the models with R_a^2 and F-statistic, testing whether the models fit data better than the null models, and verified that the final model presents the least value of Akaike Information Criterion (AIC) and Bayesian Information Criterion (BIC). We also confirmed model assumptions, including the homoscedasticity and normality of the model residuals. To further investigate the causal framework

rather than the simple correlation between the response variable and the parasitic diseases, the prevalence of parasitic diseases, which showed significant results in the final model, was analyzed as a continuous variable using univariable regression.

Results

Observed patterns through countries

The cumulative number of COVID-19 confirmed cases from March 2020 to October 2021 was 246,594,191, while the associated deaths have risen to 4,998,784 worldwide [3]. Among 202 countries included in the data, the countries in the Americas (AMR) showed the highest rates of COVID-19 incidence, followed by Europe (EUR), Southeast Asia (SEAR), East Mediterranean (EMR), Western Pacific (WPR), and Africa (AFR) (Fig 1). This trend has been consistent since the onset of the disease. The distribution of parasitic diseases was also heterogeneous across regions (Fig 2). The countries endemic for malaria (21.2 %, 42/175), schistosomiasis (22.3 %, 45/172), or STHs (26.3 %, 52/179) are distributed mainly in AFR and WPR, the regions least affected by COVID-19.

Fig 2. Differential distribution of COVID-19 and parasitic diseases. (A) COVID-19; (B) malaria; (C) schistosomiasis; (D) soil-transmitted helminthiasis

Linear regression models

A crude univariable regression analysis revealed characteristics among countries and predictive variables that were significantly associated with the risk of COVID-19 infection (Table 3). The countries with prevalent parasitic diseases presented relatively lower COVID-19 incidence rates. In the case of confounding factors, countries with a higher prevalence of tuberculosis, higher surface temperature, and later onset of COVID-19 also showed lower COVID-19 incidence rates. In contrast, the proportion of the population over 65, GDP per capita, current health expenditure per GDP, the degree of urbanization, the proportion of death by non-communicable diseases, Human Development Index, mean years of schooling, and the percentage of the population fully vaccinated with COVID-19 vaccine were positively associated with the incidence rates of COVID-19.

Table 3. Univariable analyses of predictive variables and root-transformed COVID-19 incidence rates. (changed)

Variable	Number of countries examined (%)[1]	Coefficient (95% CI)	SE of coefficient	Coefficient (95% CI) /bootstrapping	SE of coefficient /bootstrapping	*p*-value of coefficient[2]	ΔR^2
**Malaria (in %)**							
Non-endemic	156 (78.79)	Reference		Reference			
Endemic	42 (21.21)	-2.29 (-2.89, -1.69)	0.30	-2.29 (-2.64, -1.86)	0.19	< 0.001	0.23
**Schistosomiasis (in %)**							
Non-endemic	157 (77.73)	Reference		Reference			
Endemic	45 (22.27)	-2.19 (-2.82, -1.56)	0.31	-2.19 (-2.63, -1.75)	0.30	< 0.001	0.19
**STHs (in %)**							
Non-endemic	146 (73.74)	Reference		Reference			
Endemic	52 (26.26)	-1.49 (-2.13, -0.85)	0.32	-1.49 (-2.06, -0.86)	0.30	< 0.001	0.11
**Tuberculosis (in %)**							
Low (< 0.01)	55 (29.41)	Reference		Reference			
Moderate (< 0.1)	91 (48.66)	-0.99 (-1.63, -0.35)	0.32	-0.99 (-1.77, -0.61)	0.36	< 0.001	0.17
High (0.1 ≤)	41 (21.93)	-2.38 (-3.16, -1.61)	0.39	-2.38 (-3.11, -1.72)	0.34	< 0.001	
**Population ages 65 and above (in %)**							
< 5	72 (38.30)	Reference		Reference			

< 15	70 (37.23)	2.15 (1.54, 2.75)	0.35	2.15 (1.54, 2.80)	0.34	< 0.001	0.26
15 ≤	46 (24.47)	2.28 (1.60, 2.97)	0.32	2.28 (1.74, 2.88)	0.28	< 0.001	
**Death by non-communicable diseases (in %)**							
< 60	50 (27.17)	Reference		Reference			
< 80	54 (29.35)	1.96 (1.26, 2.65)	0.35	1.96 (1.32, 2.70)	0.17	< 0.001	0.25
80 ≤	80 (43.48)	2.51 (1.87, 3.15)	0.32	2.51 (2.03, 2.96)	0.14	< 0.001	
**Diabetes (in %)**							
< 5	39 (19.31)	Reference		Reference			
< 10	103 (50.99)	1.17 (0.41, 1.93)	0.38	1.17 (0.53, 1.78)	0.31	< 0.01	0.05
10 ≤	60 (29.70)	1.13 (0.29, 1.96)	0.41	1.13 (0.38, 1.98)	0.40	< 0.01	
**GDP per capita (in $)**							
< 2000	48 (24.87)	Reference		Reference			
< 6,000	43 (22.28)	1.70 (0.99, 2.43)	0.36	1.70 (1.13, 2.36)	0.30	< 0.001	0.31
< 18,000	53 (27.46)	3.02 (2.33, 3.71)	0.34	3.02 (2.41, 3.73)	0.33	< 0.001	
18,000 ≤	49 (25.39)	2.26 (1.56, 2.96)	0.35	2.26 (1.86, 2.69)	0.21	< 0.001	
**Current health expenditure (% GDP)**							
< 5	60 (38.08)	Reference		Reference			
< 8	81 (43.32)	1.59 (0.94, 2.24)	0.32	1.59 (0.94, 2.24)	0.27	< 0.001	0.14

8 ≤	46 (24.60)	1.74 (1.01, 2.49)	0.37	1.74 (1.01, 2.49)	0.35	< 0.001	
**Average surface temperature (in ℃)**							
Polar-Microthermal (< 18)	66 (33.67)	Reference		Reference			
Temperate (< 26)	60 (30.61)	-1.25 (-1.93, -0.51)	0.36	-1.25 (-1.85, -0.55)	0.33	< 0.001	0.11
Tropical (≤ 26)	70 (35.71)	-1.27 (-1.96, -0.58)	0.34	-1.27 (-1.88, -0.44)	0.36	< 0.001	
**Urban population (in %)**							
< 50	67 (33.17)	Reference		Reference			
< 75	72 (35.64)	1.91 (1.27, 2.56)	0.32	1.91 (1.33, 2.60)	0.32	< 0.001	0.17
75 ≤	63 (31.19)	1.69 (1.02, 2.35)	0.33	1.69 (1.15, 2.24)	0.27	< 0.001	
**Fully vaccinated with COVID-vaccine (in %)**							
< 30	86 (43.21)	Reference		Reference			
< 60	61 (30.65)	2.20 (1.58, 2.82)	0.31	2.20 (1.55, 2.82)	0.32	< 0.001	0.30
60 ≤	52 (26.13)	1.27 (0.67, 1.92)	0.33	1.27 (0.71, 2.11)	0.33	< 0.001	
**Population density (per km²)**							
< 50	67 (33.83)	Reference		Reference			
< 120	59 (29.80)	0.36 (-0.37, 1.65)	0.37	0.36 (-0.36, 1.45)	0.34	0.34	0.02
120 ≤	72 (36.36)	0.20 (-0.50, 0.98)	0.35	0.20 (-0.47, 0.88)	0.36	0.57	
**Human development index**							

< 0.6	46 (24.60)	Reference		Reference			
< 0.8	76 (40.64)	2.43 (1.80, 3.06)	0.32	2.43 (1.93, 2.97)	0.32	< 0.001	0.31
0.8 ≤	65 (34.76)	2.85 (2.20, 3.51)	0.33	2.85 (2.48, 3.33)	0.33	< 0.001	
**Duration (in days)**							
< 70	104 (52.26)	Reference		Reference			
70 ≤	95 (47.74)	-1.34 (-1.89, -0.79)	0.28	-1.34 (-1.89, -0.78)	0.27	< 0.001	0.19
**Mean years of schooling (in years)**							
< 7	58 (31.35)	Reference		Reference			
< 10	50 (27.03)	1.59 (1.04, 1.77)	0.33	1.59 (1.11, 1.67)	0.28	< 0.001	0.30
10 ≤	77 (41.62)	1.97 (1.54, 2.20)	0.29	1.97 (1.64, 2.19)	0.31	< 0.001	

[1]Percentages reflect cases without missing values

[2]The level of statistical significance is 0.05

A multivariable model with parasitic diseases including malaria, schistosomiasis, and STHs was analyzed as a fundamental model (Table 4). COVID-19 incidence rates were significantly lower in countries endemic for malaria (β coefficient = -1.38; 95 % CI of -2.33 to -0.41; $p < 0.01$). The countries where schistosomiasis or STHs are prevailing, on the other hand, were not revealed to have a significant association with COVID-19 incidence rates, though the coefficients were both negative for schistosomiasis (β coefficient = -0.84; 95 % CI of -1.76 to 0.15; $p = 0.09$) and STHs (β coefficient = -0.58; 95 % CI of -1.20 to 0.01; $p = 0.05$).

Table 4. Summary of associations between parasitic disease variables and root-transformed COVID-19 incidence rates. (changed)

**Variable**[1]	**Coefficient (95% CI)**	**SE of coefficient**	**Coefficient (95% CI) /bootstrapping**	**SE of coefficient /bootstrapping**	***p*-value of coefficient**	**Adjusted ΔR^2**	***p*-value of ΔR^2**
**Malaria (in %)**							
Non-endemic	Reference		Reference				
Endemic	-1.38 (-2.33, -0.41)	0.48	-1.38 (-2.06, -0.63)	0.36	< 0.01		
**Schistosomiasis (in %)**							
Non-endemic	Reference		Reference			0.24	< 0.001
Endemic	-0.84 (-1.76, 0.15)	0.48	-0.84 (-1.57, -0.04)	0.38	0.09		
**STHs (in %)**							
Non-endemic	Reference		Reference				
Endemic	-0.58 (-1.20, 0.01)	0.32	-0.58 (-1.15, 0.02)	0.32	0.05		

[1]Interaction terms were not significant

After screening significant variables using univariable analysis, variables with pairwise correlation coefficients between -0.7 and 0.7 were comprised for further multivariable linear regression (S1 Fig). The saturated model had the least value of AIC and BIC (S1 Table). Since the AIC and BIC significantly increased when the parasitic disease variables were removed from the model, it could be concluded that the variables explain a considerable level of variation in the saturated model. A total of 165 countries were retained in the model, excluding missing values. Table 5 summarizes key results from the multivariable regression model of imputed predictive variables for the response variable, COVID-19 incidence rates. In the model, the malaria-endemic countries presented significantly lower COVID-19 incidence rates (β coefficient = -0.73; 95 % CI of -1.46 to -0.10; $p < 0.05$) even adjusting for confounding variables, the timing of the disease eruption (duration variable), the proportion of the population over 65, and GDP per capita, while schistosomiasis (β coefficient = -0.30; 95 % CI of -1.02 to 0.40; $p = 0.39$) and STHs (β coefficient = -0.14; 95 % CI of -0.67 to 0.39; $p = 0.59$) were not significantly associated with the response variable. In addition, the model examined GDP per capita to have significant associations with COVID-19 incidence rates. In the univariable regression model with the prevalence of malaria as a predictor, and COVID-19 incidence rates as an outcome, the outcome variable monotonically decreased as the predictor increased (β coefficient = -0.02; 95 % CI of -0.04 to -0.01; $p < 0.01$) (Fig 3).

Fig 3. Scatter plot diagram of the malaria prevalence and case incidence rates of COVID-19 for malaria-endemic countries.

Table 5. Summary of multivariable linear regression, including parasite variables of interest and potential confounders. (changed)

Variable	Coefficient (95% CI)	SE of coefficient	Coefficient (95% CI) /bootstrapping	SE of coefficient /bootstrapping	*p*-value of coefficient	Adjusted ΔR^2	*p*-value of ΔR^2
**Malaria**							
Non-endemic	Reference		Reference				
Endemic	-0.73 (-1.46, -0.10)	0.36	-0.73 (-1.42, -0.12)	0.34	< 0.05		
**Schistosomiasis**							
Non-endemic	Reference		Reference				
Endemic	-0.30 (-1.02, 0.40)	0.36	-0.30 (-1.06, 0.34)	0.36	0.39		
**STHs**							
Non-endemic	Reference		Reference				
Endemic	-0.14 (-0.67, 0.39)	0.26	-0.14 (-0.64, 0.45)	0.28	0.59		
**Duration (in days)**							
< 70	Reference		Reference			0.49	< 0.001
70 ≤	-0.40 (-0.87, 0.07)	0.24	-0.40 (-0.88, 0.09)	0.29	0.11		
**Population ages 65 and above (in %)**							
< 5	Reference		Reference				
< 15	0.17(-0.41, 0.74)	0.29	0.17(-0.45, 0.80)	0.33	0.56		
15 ≤	0.35 (-0.38, 1.04)	0.35	0.35 (-0.35, 1.02)	0.36	0.36		
**GDP per capita (in $)**							

< 2000	Reference		Reference		
< 6,000	0.88 (0.25, 1.51)	0.31	0.88 (0.26, 1.58)	0.32	< 0.001
< 18,000	1.86 (1.12, 2.59)	0.37	1.86 (1.26, 2.64)	0.34	< 0.001
18,000 ≤	0.96 (0.12, 1.80)	0.41	0.96 (0.22, 1.78)	0.39	< 0.05

Discussion

We performed an ecological study to reveal crucial factors that may explain the worldwide disparity of SARS-CoV-2 epidemics, focusing on the significance of exposure to endemic parasitic infections. We identified that malaria prevalence is an essential factor explaining variation in observed case incidence of COVID-19 at the national level, with a significant association retained even when possible confounders were adjusted. Potential associations of schistosomiasis and STHs with SARS-CoV-2 infection appeared unlikely, from this study, to be critical factors in the heterogeneity in SARS-CoV-2 epidemic trends. Overall, while we emphasize that causal inference cannot be concluded due to incomplete data and hidden confounders, we discovered that the prevalence of malaria has a consistent association that indicates its impact on COVID-19 epidemics, indicating that further research is needed.

The malaria prevalence was the only measure of parasitic infection that consistently showed a strong negative association with COVID-19 incidence rates. One plausible biological mechanism might be the variable distribution of the polymorphisms associated with angiotensin-converting enzyme 2 (ACE2). A type I transmembrane amino-peptidase, ACE2, is anchored at the surface of cells of the heart, kidneys, gastrointestinal system, blood vessels, and type II alveolar cells of the lungs [45]. Angiotensin II (ANG II), the substrate for ACE2, was reported to impair *Plasmodium* development, directly disturbing the protozoa membrane and protecting the human host from malaria [46,47]. The presence of genetic deletion/insertion (D/I) and C1173T substitution polymorphisms are linked to changes in the concentration of ACE2, reducing ACE2 expression in the dominance of the D allele and T allele, respectively [46]. Subsequently, ANG II plasma levels are increased due to the reduction of ACE2, alleviating the clinical course of malaria, which may be a powerful driver of natural selection in malaria-endemic regions. SARS-CoV-2 requires the ACE2 receptor to enter the host cells, especially the type II alveolar cells [48]. The downregulation of ACE2 receptors due to the polymorphisms may result in a "protective" effect influencing the cell entry of SARS-CoV-2 [49]. Therefore, this mutual influence may explain the differential distribution of COVID-19 among countries, though additional genetic studies are needed to better understand the protective role of ACE2 polymorphisms.

Another hypothesis proposed from the literature was about the cross-reactivity of interferons and the neutralizing antibodies. The CD-147 receptor, expressed on several blood cells and immune cells, is a common entryway for both coronavirus and malarial *Plasmodium* to invade host cells [50]. Reports from previous studies have shown that lymphocytes produced interferons as an immune response to infection by some strains of *Plasmodium*; since the pathogens share the receptor for invasion, these interferons have a protective effect against the coronaviruses responsible for MERS, SARS, and

COVID-19 [51-53]. The contribution of CD-147 blockers, Azithromycin, to preventing coronavirus infection also serves as evidence for the hypothesis [50]. Moreover, prior exposure to malaria enhances the production of *Plasmodium*-specific IgG antibodies targeting glycosylphosphatidylinositol (GPI) molecules, also capable of interacting with various glycoproteins (GPs) of SARS-CoV-2, which may confer some resistance against the coronavirus infection [54]. Although previous malaria infections cannot fully protect individuals from the disease, the severity of clinical presentation in individuals who repeatedly encountered malaria infections is milder than in "non-immune" subjects [55], suggesting that this scenario may occur in the cases of coronavirus infection.

The problems in early diagnosis due to the similar clinical features between malaria and COVID‑19 may also have caused the lower number of reported COVID-19 cases in malaria-endemic regions. The first step to identifying a COVID-19 case is symptomatic screening. Early symptoms of SARS-CoV-2 infection such as fever, difficulty in breathing, and fatigue could be confused with symptoms of malaria, leading to the misdiagnosis of COVID-19 patients as malaria and vice versa [46,49]. Patients with clinical symptoms may be tested for *Plasmodium* infection when they actually have COVID-19 and then ignored due to a negative result. Therefore, especially in malaria-endemic areas, it would be recommended to double-screen patients with suspected symptoms of both malaria and COVID-19.

Helminth infection has various immunomodulatory effects, leading to enhanced resistance to some pathogens, increased susceptibility to others, and variations in the severity of autoimmune, allergic, and inflammatory diseases [23,56]. Moreover, several studies proposed immunomodulatory properties of helminths, which may result in enhanced antiviral mechanisms, such as the role of *Fasciola hepatica* products in the host [16], a relatively milder course of COVID-19 in *Wuchereria bancrofti* and SARS-CoV-2 co-infection [24], lower risk of respiratory viral infections in mice with schistosomiasis [57], limited inflammatory damage induced by influenza virus in *Trichinella spiralis* infected hosts [58], and indirect protective strategy through changes of diversity and composition of the microbiome [15]. It has also been suggested that helminth infection may account for better tolerance of viral infections, including SARS-CoV-2 [23,59]. However, some helminths may exacerbate the clinical course of viral infection. As Siles-Lucas et al. [16] discussed, *Heligmosomoides polygyrus* and *T. spiralis* infection in mice could enhance viral infections; *S. mansoni* induces a pro-inflammatory response in the early stages of the infection [60], which may lead to synergistic epidemics and more severe manifestations of COVID-19. Overall, potential associations of schistosomiasis and STHs with COVID-19 appeared unlikely, at least based on this study, to affect the differential distribution of COVID-19. Analysis with aggregated data may be insufficiently granular to discover a potential influence of endemic infections. A previous study [61] reported that within-country variation in an immunological profile associated

with changes in exposure to diseases could be detected. Our analysis at the national level could not fully reflect this heterogeneity.

Our findings present that GDP per capita, the measure of income, is significantly associated with the variable distribution of COVID-19. According to a large body of research, this factor leads to better health outcomes by allowing for improved health infrastructure and the funding of effective containment strategies such as social distancing [2,62,63]. However, it warrants further research to determine why “richer” countries with the capacity to control the spread of the disease effectively have higher incidence rates of COVID-19 in the multivariable model. As described in a previous study [2], the variable that presents the duration of the pandemic in each country adjusted the effect of disparities in the timing of disease onset, confirming whether the difference in COVID-19 distribution between countries is simply because each country is in the different phases of the pandemic. On the other hand, the proportion of the population aged 65 and above was not significantly associated with the transmission of COVID-19, based on this study. The factor was suggested as an essential demographic indicator capturing population aging and affecting the morbidity of COVID-19 in previous literature [2,9,39], which contrasts with the results of this study. Although this result may be due to the limitation of ecological research and incomplete data, one possible hypothesis is the presence of endogenous behavioral reactions. Since they are more vulnerable, older people are more likely to be cautious in implementing social distancing measures and safety precautions than others, which may finally reduce their greater infection risk, resulting in no statistical significance in an empirical analysis.

In April 2020, because of the COVID-19 pandemic, WHO declared a general recommendation to disrupt intervention programs for Neglected Tropical Diseases (NTDs). The impact of this interruption, paired with COVID-19, will reverberate for a year. The complicated effects of the COVID-19 pandemic might result in an additional 200 million malaria infections and 380,000 associated deaths in sub-Saharan Africa [64]. Similarly, it is predicted to require extra time to cope with increased infection levels of two other infectious diseases, schistosomiasis and STHs, whose prevention programs were interrupted by the efforts to reduce the COVID-19 spread [25]. The current global priority of protecting people from attaining the SARS-CoV-2 infection, resulting in the interruption of activities of the other health sectors, makes the already vulnerable part of the population even more fragile and “neglected.”

Despite the strengths of this study, there are several limitations to be noted. Related to the ecological design, the results of this study might be biased due to misclassification of the exposure to endemic diseases. Although within-country variation is frequently observed in the distribution of parasitic diseases, including malaria, schistosomiasis, and STHs, the aggregated nature of the data could not fully

reflect this heterogeneity. Therefore, individual-level data with precise locations are needed to assess the exact correlation between the coronavirus and indigenous diseases. The other significant limitation is the misclassification of COVID-19 cases due to testing capacity and surveillance quality, likely to vary among countries. Due to the differences in health infrastructure and testing policy, the data may obscure the accurate size of the diseases [5]. Studies have revealed significantly under-reported cases and associated deaths in several countries, especially in low-income countries [65]. Even though we conceptualized the true number of the infections based on TPR to adjust possible biases in the under-reporting of the COVID-19 cases, we cannot conclude that our study has perfectly precluded them. Thus, our results should be cautiously interpreted. We also want to point out that we might not have adjusted for all confounding variables, though we attempted to include demographic, socioeconomic, and geographic variables suggested in previous studies. Our study is based on an analysis describing a still evolving and imperfectly explored epidemic, only considering the scientific understanding of the disease at the time. Therefore, due to these unknown factors, our study could not deal with all factors that influence the spread of COVID-19 and residual confounding.

This study aimed to assess the role of parasitic diseases in explaining the spread of COVID-19 and to understand why the disease is progressing at a slower pace in LMICs. Malaria prevalence appears to be an essential element associated with the epidemics of SARS-CoV-2, even after potential confounding factors were adjusted. Our analyses suggest significantly lower COVID-19 incidence rates in malaria-endemic regions, showing a negative dose-response relationship. However, our findings do not conclusively support the causal inference of endemic diseases to SARS-CoV-2 transmission. Although we obtained results indicating that malaria infection has a kind of "protective effect" against coronavirus, further investigation at the subnational or individual level should be conducted on the interaction of SARS-CoV-2 with various human infecting parasites to prevent unexpected harm. The COVID-19 pandemic will have long-term social, economic, and health consequences worldwide. The researchers are responsible for advocating for the resume of intervention programs for malaria and NTDs on the global health agenda. When the world is prioritizing the historical pandemic, this moment could be pivotal to strengthening the health systems that prevent malaria and NTDs.

Acknowledgments

We have no acknowledgment related to this work.

Conflicts of interest

We have no conflict of interest related to this work.

1. Makoni M. COVID-19 in Africa: half a year later. Lancet Infect Dis. 2020;20(10):1127. doi: 10.1016/S1473-3099(20)30708-8. PubMed PMID: 32979326; PubMed Central PMCID: PMCPMC7511168.
2. Nguimkeu P, Tadadjeu S. Why is the number of COVID-19 cases lower than expected in Sub-Saharan Africa? A cross-sectional analysis of the role of demographic and geographic factors. World Dev. 2021;138:105251. Epub 20201021. doi: 10.1016/j.worlddev.2020.105251. PubMed PMID: 33106726; PubMed Central PMCID: PMCPMC7577660.
3. WHO Coronavirus Disease (COVID-19) Dashboard. Geneva, Switzerland: World Health Organization; 2021 [cited 2021 November 1]. Available from: https://covid19.who.int/.
4. Nkengasong JN, Mankoula W. Looming threat of COVID-19 infection in Africa: act collectively, and fast. Lancet. 2020;395(10227):841-2. Epub 20200227. doi: 10.1016/S0140-6736(20)30464-5. PubMed PMID: 32113508; PubMed Central PMCID: PMCPMC7124371.
5. Favas C, Jarrett P, Ratnayake R, Watson OJ, Checchi F. Country differences in transmissibility, age distribution and case-fatality of SARS-CoV-2: a global ecological analysis. Int J Infect Dis. 2022;114:210-8. Epub 20211106. doi: 10.1016/j.ijid.2021.11.004. PubMed PMID: 34749011; PubMed Central PMCID: PMCPMC8571103.
6. Mbow M, Lell B, Jochems SP, Cisse B, Mboup S, Dewals BG, et al. COVID-19 in Africa: Dampening the storm? Science. 2020;369(6504):624-6. doi: 10.1126/science.abd3902. PubMed PMID: 32764055.
7. Massinga Loembe M, Tshangela A, Salyer SJ, Varma JK, Ouma AEO, Nkengasong JN. COVID-19 in Africa: the spread and response. Nat Med. 2020;26(7):999-1003. doi: 10.1038/s41591-020-0961-x. PubMed PMID: 32528154.
8. Fang L, Karakiulakis G, Roth M. Are patients with hypertension and diabetes mellitus at increased risk for COVID-19 infection? Lancet Respir Med. 2020;8(4):e21. Epub 20200311. doi: 10.1016/S2213-2600(20)30116-8. PubMed PMID: 32171062; PubMed Central PMCID: PMCPMC7118626.
9. Davies NG, Klepac P, Liu Y, Prem K, Jit M, group CC-w, et al. Age-dependent effects in the transmission and control of COVID-19 epidemics. Nat Med. 2020;26(8):1205-11. Epub 20200616. doi: 10.1038/s41591-020-0962-9. PubMed PMID: 32546824.
10. Driggin E, Madhavan MV, Bikdeli B, Chuich T, Laracy J, Biondi-Zoccai G, et al. Cardiovascular Considerations for Patients, Health Care Workers, and Health Systems During the COVID-19 Pandemic. J Am Coll Cardiol. 2020;75(18):2352-71. Epub 20200319. doi: 10.1016/j.jacc.2020.03.031. PubMed PMID: 32201335; PubMed Central PMCID: PMCPMC7198856.
11. Poel EV, O'Donnell O, Doorslaer EV. Is there a health penalty of China's rapid urbanization? Health Econ. 2012;21(4):367-85. Epub 20110221. doi: 10.1002/hec.1717. PubMed PMID: 21341344.

12. Speich B, Croll D, Furst T, Utzinger J, Keiser J. Effect of sanitation and water treatment on intestinal protozoa infection: a systematic review and meta-analysis. Lancet Infect Dis. 2016;16(1):87-99. Epub 20150921. doi: 10.1016/S1473-3099(15)00349-7. PubMed PMID: 26404667.
13. Herricks JR, Hotez PJ, Wanga V, Coffeng LE, Haagsma JA, Basanez MG, et al. The global burden of disease study 2013: What does it mean for the NTDs? PLoS Negl Trop Dis. 2017;11(8):e0005424. Epub 20170803. doi: 10.1371/journal.pntd.0005424. PubMed PMID: 28771480; PubMed Central PMCID: PMCPMC5542388.
14. World Malaria Report 2019. Geneva, Switzerland: World Health Organization, 2019.
15. Wolday D, Tasew G, Amogne W, Urban B, Schallig HD, Harris V, et al. Interrogating the Impact of Intestinal Parasite-Microbiome on Pathogenesis of COVID-19 in Sub-Saharan Africa. Front Microbiol. 2021;12:614522. Epub 20210416. doi: 10.3389/fmicb.2021.614522. PubMed PMID: 33935986; PubMed Central PMCID: PMCPMC8086792.
16. Siles-Lucas M, Gonzalez-Miguel J, Geller R, Sanjuan R, Perez-Arevalo J, Martinez-Moreno A. Potential Influence of Helminth Molecules on COVID-19 Pathology. Trends Parasitol. 2021;37(1):11-4. Epub 20201015. doi: 10.1016/j.pt.2020.10.002. PubMed PMID: 33153921; PubMed Central PMCID: PMCPMC7561300.
17. Ryan SM, Eichenberger RM, Ruscher R, Giacomin PR, Loukas A. Harnessing helminth-driven immunoregulation in the search for novel therapeutic modalities. PLoS Pathog. 2020;16(5):e1008508. Epub 20200514. doi: 10.1371/journal.ppat.1008508. PubMed PMID: 32407385; PubMed Central PMCID: PMCPMC7224462.
18. Burrows K, Ngai L, Wong F, Won D, Mortha A. ILC2 Activation by Protozoan Commensal Microbes. Int J Mol Sci. 2019;20(19). Epub 20190930. doi: 10.3390/ijms20194865. PubMed PMID: 31574995; PubMed Central PMCID: PMCPMC6801642.
19. McSorley HJ, Maizels RM. Helminth infections and host immune regulation. Clin Microbiol Rev. 2012;25(4):585-608. doi: 10.1128/CMR.05040-11. PubMed PMID: 23034321; PubMed Central PMCID: PMCPMC3485755.
20. Maizels RM, McSorley HJ. Regulation of the host immune system by helminth parasites. J Allergy Clin Immunol. 2016;138(3):666-75. Epub 20160729. doi: 10.1016/j.jaci.2016.07.007. PubMed PMID: 27476889; PubMed Central PMCID: PMCPMC5010150.
21. Bradbury RS, Piedrafita D, Greenhill A, Mahanty S. Will helminth co-infection modulate COVID-19 severity in endemic regions? Nat Rev Immunol. 2020;20(6):342. doi: 10.1038/s41577-020-0330-5. PubMed PMID: 32358579; PubMed Central PMCID: PMCPMC7193760.
22. Abdoli A, Ardakani HM. Helminth infections and immunosenescence: The friend of my enemy. Exp Gerontol. 2020;133:110852. Epub 20200130. doi: 10.1016/j.exger.2020.110852. PubMed PMID: 32007545.
23. Fonte L, Acosta A, Sarmiento ME, Ginori M, Garcia G, Norazmi MN. COVID-19 Lethality in Sub-Saharan Africa and Helminth Immune Modulation. Front Immunol. 2020;11:574910. Epub 20201008. doi: 10.3389/fimmu.2020.574910. PubMed PMID: 33117371; PubMed Central PMCID: PMCPMC7578247.
24. Mohamed MFH, Mohamed SF, Yousaf Z, Kohla S, Howady F, Imam Y. COVID-19 unfolding filariasis: The first case of SARS-CoV-2 and Wuchereria bancrofti coinfection. PLoS Negl Trop

Dis. 2020;14(11):e0008853. Epub 20201109. doi: 10.1371/journal.pntd.0008853. PubMed PMID: 33166283; PubMed Central PMCID: PMCPMC7652262.

25. Gluchowska K, Dzieciatkowski T, Sedzikowska A, Zawistowska-Deniziak A, Mlocicki D. The New Status of Parasitic Diseases in the COVID-19 Pandemic-Risk Factors or Protective Agents? J Clin Med. 2021;10(11). Epub 20210607. doi: 10.3390/jcm10112533. PubMed PMID: 34200502; PubMed Central PMCID: PMCPMC8200987.
26. Gutman JR, Lucchi NW, Cantey PT, Steinhardt LC, Samuels AM, Kamb ML, et al. Malaria and Parasitic Neglected Tropical Diseases: Potential Syndemics with COVID-19? Am J Trop Med Hyg. 2020;103(2):572-7. Epub 20200601. doi: 10.4269/ajtmh.20-0516. PubMed PMID: 32484155; PubMed Central PMCID: PMCPMC7410484.
27. Wu D, Yang XO. TH17 responses in cytokine storm of COVID-19: An emerging target of JAK2 inhibitor Fedratinib. J Microbiol Immunol Infect. 2020;53(3):368-70. Epub 20200311. doi: 10.1016/j.jmii.2020.03.005. PubMed PMID: 32205092; PubMed Central PMCID: PMCPMC7156211.
28. Vatsalya V, Li F, Frimodig JC, Gala KS, Srivastava S, Kong M, et al. Therapeutic Prospects for Th-17 Cell Immune Storm Syndrome and Neurological Symptoms in COVID-19: Thiamine Efficacy and Safety, In-vitro Evidence and Pharmacokinetic Profile. medRxiv. 2020. Epub 20200825. doi: 10.1101/2020.08.23.20177501. PubMed PMID: 32869036; PubMed Central PMCID: PMCPMC7457607.
29. Liao YC, Liang WG, Chen FW, Hsu JH, Yang JJ, Chang MS. IL-19 induces production of IL-6 and TNF-alpha and results in cell apoptosis through TNF-alpha. J Immunol. 2002;169(8):4288-97. doi: 10.4049/jimmunol.169.8.4288. PubMed PMID: 12370360.
30. Fitzpatrick P. The challenges of international comparisons of COVID-19. Ir J Med Sci. 2021;190(2):483-4. Epub 20200921. doi: 10.1007/s11845-020-02370-9. PubMed PMID: 32955699; PubMed Central PMCID: PMCPMC7502653.
31. Tuite AR, Ng V, Rees E, Fisman D. Estimation of COVID-19 outbreak size in Italy. Lancet Infect Dis. 2020;20(5):537. Epub 20200319. doi: 10.1016/S1473-3099(20)30227-9. PubMed PMID: 32199494; PubMed Central PMCID: PMCPMC7271205.
32. Giordano G, Blanchini F, Bruno R, Colaneri P, Di Filippo A, Di Matteo A, et al. Modelling the COVID-19 epidemic and implementation of population-wide interventions in Italy. Nat Med. 2020;26(6):855-60. Epub 20200422. doi: 10.1038/s41591-020-0883-7. PubMed PMID: 32322102; PubMed Central PMCID: PMCPMC7175834.
33. Kuster AC, Overgaard HJ. A novel comprehensive metric to assess effectiveness of COVID-19 testing: Inter-country comparison and association with geography, government, and policy response. PLoS One. 2021;16(3):e0248176. Epub 20210305. doi: 10.1371/journal.pone.0248176. PubMed PMID: 33667280; PubMed Central PMCID: PMCPMC7935311.
34. Fraser C, Donnelly CA, Cauchemez S, Hanage WP, Van Kerkhove MD, Hollingsworth TD, et al. Pandemic potential of a strain of influenza A (H1N1): early findings. Science. 2009;324(5934):1557-61. Epub 20090511. doi: 10.1126/science.1176062. PubMed PMID: 19433588; PubMed Central PMCID: PMCPMC3735127.
35. Nishiura H, Klinkenberg D, Roberts M, Heesterbeek JA. Early epidemiological assessment of the virulence of emerging infectious diseases: a case study of an influenza pandemic. PLoS

One. 2009;4(8):e6852. Epub 20090831. doi: 10.1371/journal.pone.0006852. PubMed PMID: 19718434; PubMed Central PMCID: PMCPMC2729920.

36. The Global Health Observatory. Geneva, Switzerland: World Health Organization; [cited 2021 October 1]. Available from: https://www.who.int/data/gho/data/indicators.

37. World Development Indicators. Washington, USA: The World Bank; [cited 2021 October 1]. Available from: https://databank.worldbank.org/home.

38. World Health O. Coronavirus disease 2019 (COVID-19): situation report, 72. Geneva: World Health Organization, 2020 2020-04-01. Report No.

39. Zhou F, Yu T, Du R, Fan G, Liu Y, Liu Z, et al. Clinical course and risk factors for mortality of adult inpatients with COVID-19 in Wuhan, China: a retrospective cohort study. Lancet. 2020;395(10229):1054-62. Epub 20200311. doi: 10.1016/S0140-6736(20)30566-3. PubMed PMID: 32171076; PubMed Central PMCID: PMCPMC7270627.

40. Stein R, Wroth C, Hurt A. U.S. Coronavirus Testing Still Falls Short. How's Your State Doing?2020 [cited 2022 01 July]. Available from: https://www.npr.org/sections/health-shots/2020/05/07/851610771/u-s-coronavirus-testing-still-falls-short-hows-your-state-doing.

41. Team RC. R: A language and environment for statistical computing. R Foundation for Statistical Computing, Vienna, Austria. 2021.

42. Canty A, Ripley BD. boot: Bootstrap R (S-Plus) Functions. R package version 1.3-28 ed2021.

43. Davison AC, Hinkley DV. Bootstrap Methods and their Application. Cambridge: Cambridge University Press; 1997.

44. Blanchet FG, Legendre P, Borcard D. Forward selection of explanatory variables. Ecology. 2008;89(9):2623-32. doi: 10.1890/07-0986.1. PubMed PMID: 18831183.

45. Zhou P, Yang XL, Wang XG, Hu B, Zhang L, Zhang W, et al. A pneumonia outbreak associated with a new coronavirus of probable bat origin. Nature. 2020;579(7798):270-3. Epub 20200203. doi: 10.1038/s41586-020-2012-7. PubMed PMID: 32015507; PubMed Central PMCID: PMCPMC7095418.

46. Hussein MIH, Albashir AAD, Elawad OAMA, Homeida A. Malaria and COVID-19: unmasking their ties. Malaria Journal. 2020;19(1):457. doi: 10.1186/s12936-020-03541-w.

47. Silva LS, Silva-Filho JL, Caruso-Neves C, Pinheiro AA. New Concepts in Malaria Pathogenesis: The Role of the Renin-Angiotensin System. Front Cell Infect Microbiol. 2015;5:103. Epub 20160107. doi:10.3389/fcimb.2015.00103. PubMed PMID: 26779452; PubMed Central PMCID: PMCPMC4703750.

48. Hoffmann M, Kleine-Weber H, Krüger N, Müller M, Drosten C, Pöhlmann S. The novel coronavirus 2019 (2019-nCoV) uses the SARS-coronavirus receptor ACE2 and the cellular protease TMPRSS2 for entry into target cells. bioRxiv. 2020:2020.01.31.929042. doi: 10.1101/2020.01.31.929042.

49. Di Gennaro F, Marotta C, Locantore P, Pizzol D, Putoto G. Malaria and COVID-19: Common and Different Findings. Trop Med Infect Dis. 2020;5(3). Epub 20200906. doi: 10.3390/tropicalmed5030141. PubMed PMID: 32899935; PubMed Central PMCID: PMCPMC7559940.

50. Ulrich H, Pillat MM. CD147 as a Target for COVID-19 Treatment: Suggested Effects of Azithromycin and Stem Cell Engagement. Stem Cell Rev Rep. 2020;16(3):434-40. doi: 10.1007/s12015-020-09976-7. PubMed PMID: 32307653; PubMed Central PMCID: PMCPMC7167302.

51. King T, Lamb T. Interferon-gamma: The Jekyll and Hyde of Malaria. PLoS Pathog. 2015;11(10):e1005118. Epub 20151001. doi: 10.1371/journal.ppat.1005118. PubMed PMID: 26426121; PubMed Central PMCID: PMCPMC4591348.
52. Strayer DR, Dickey R, Carter WA. Sensitivity of SARS/MERS CoV to interferons and other drugs based on achievable serum concentrations in humans. Infect Disord Drug Targets. 2014;14(1):37-43. doi: 10.2174/1871526514666140713152858. PubMed PMID: 25019238.
53. Fauci AS, Lane HC, Redfield RR. Covid-19 - Navigating the Uncharted. N Engl J Med. 2020;382(13):1268-9. Epub 20200228. doi: 10.1056/NEJMe2002387. PubMed PMID: 32109011; PubMed Central PMCID: PMCPMC7121221.
54. Gomes LR, Martins YC, Ferreira-da-Cruz MF, Daniel-Ribeiro CT. Autoimmunity, phospholipid-reacting antibodies and malaria immunity. Lupus. 2014;23(12):1295-8. doi: 10.1177/0961203314546021. PubMed PMID: 25228731.
55. Mendonca VR, Barral-Netto M. Immunoregulation in human malaria: the challenge of understanding asymptomatic infection. Mem Inst Oswaldo Cruz. 2015;110(8):945-55. Epub 20151211. doi: 10.1590/0074-02760150241. PubMed PMID: 26676319; PubMed Central PMCID: PMCPMC4708013.
56. Maizels RM. Regulation of immunity and allergy by helminth parasites. Allergy. 2020;75(3):524-34. Epub 20190718. doi: 10.1111/all.13944. PubMed PMID: 31187881.
57. Schwartz C, Hams E, Fallon PG. Helminth Modulation of Lung Inflammation. Trends Parasitol. 2018;34(5):388-403. Epub 20180112. doi: 10.1016/j.pt.2017.12.007. PubMed PMID: 29339033.
58. Furze RC, Hussell T, Selkirk ME. Amelioration of influenza-induced pathology in mice by coinfection with Trichinella spiralis. Infect Immun. 2006;74(3):1924-32. doi: 10.1128/IAI.74.3.1924-1932.2006. PubMed PMID: 16495568; PubMed Central PMCID: PMCPMC1418664.
59. Ssebambulidde K, Segawa I, Abuga KM, Nakate V, Kayiira A, Ellis J, et al. Parasites and their protection against COVID-19- Ecology or Immunology? medRxiv. 2020:2020.05.11.20098053. doi: 10.1101/2020.05.11.20098053.
60. Scheer S, Krempl C, Kallfass C, Frey S, Jakob T, Mouahid G, et al. S. mansoni bolsters anti-viral immunity in the murine respiratory tract. PLoS One. 2014;9(11):e112469. Epub 20141114. doi: 10.1371/journal.pone.0112469. PubMed PMID: 25398130; PubMed Central PMCID: PMCPMC4232382.
61. Mbow M, de Jong SE, Meurs L, Mboup S, Dieye TN, Polman K, et al. Changes in immunological profile as a function of urbanization and lifestyle. Immunology. 2014;143(4):569-77. doi: 10.1111/imm.12335. PubMed PMID: 24924958; PubMed Central PMCID: PMCPMC4253505.
62. Marmot M. The Influence Of Income On Health: Views Of An Epidemiologist. Health Affairs. 2002;21(2):31-46. doi: 10.1377/hlthaff.21.2.31.
63. Condliffe S, Link CR. The Relationship between Economic Status and Child Health: Evidence from the United States. American Economic Review. 2008;98(4):1605-18. doi: 10.1257/aer.98.4.1605.
64. Sherrard-Smith E, Hogan AB, Hamlet A, Watson OJ, Whittaker C, Winskill P, et al. The potential public health consequences of COVID-19 on malaria in Africa. Nat Med.

2020;26(9):1411-6. Epub 20200807. doi: 10.1038/s41591-020-1025-y. PubMed PMID: 32770167.
65. Whittaker C, Walker PGT, Alhaffar M, Hamlet A, Djaafara BA, Ghani A, et al. Under-reporting of deaths limits our understanding of true burden of covid-19. BMJ. 2021;375:n2239. doi: 10.1136/bmj.n2239.

Supporting information

S1 Fig. Variable collinearity heatmap. Values in each cell are the Pearson correlation coefficient for a given pair of variables. Coefficients close to -1 or +1 indicate high correlation, with 0 indicating minimal correlation.

S1 Table. AIC and BIC of hierarchical models.

Figure 1

Click here to access/download;Figure;Fig 1.tif

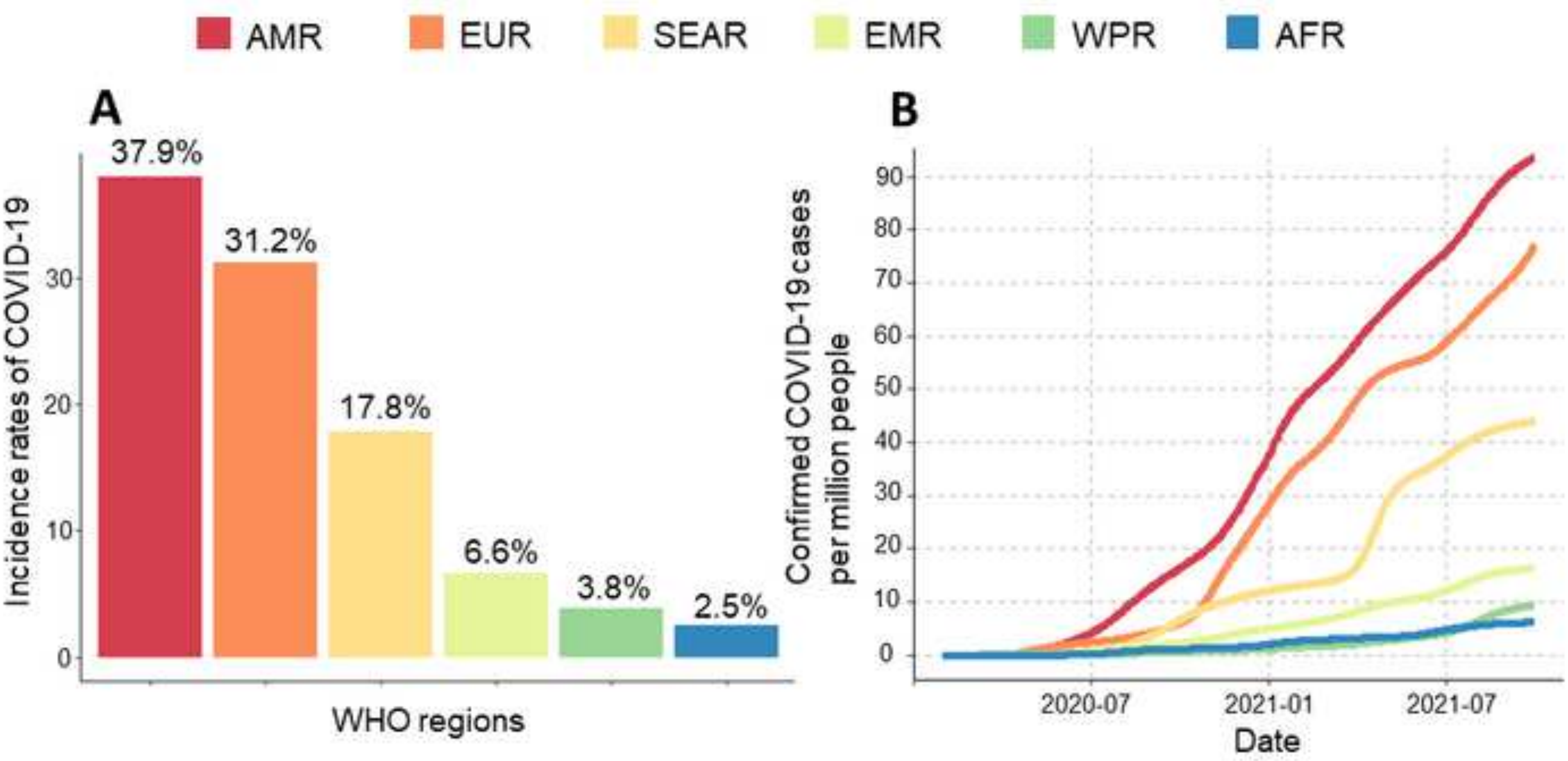

Figure 2

Click here to access/download;Figure;Fig 2.tif

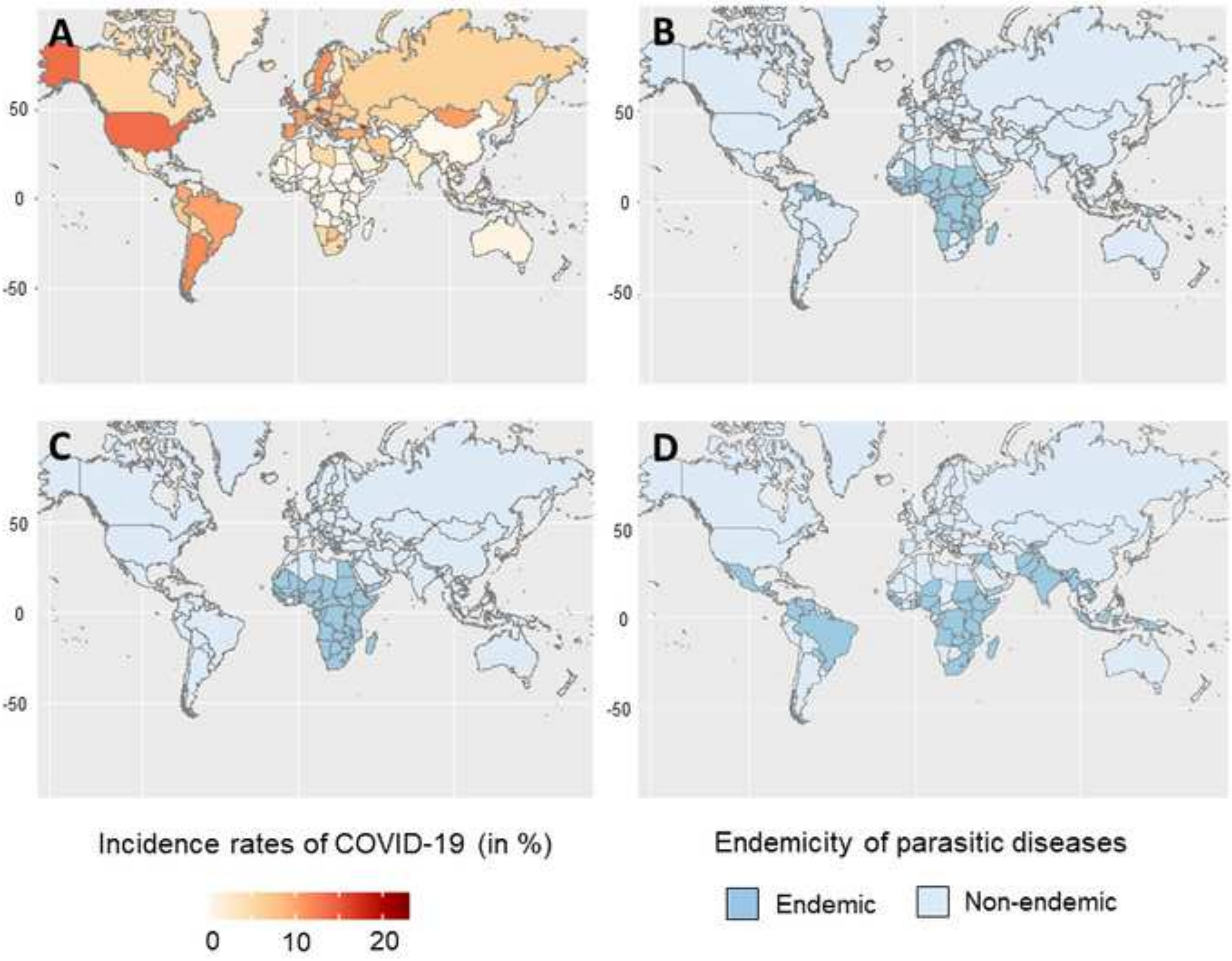

Figure 3

Click here to access/download;Figure;Fig 3.tif

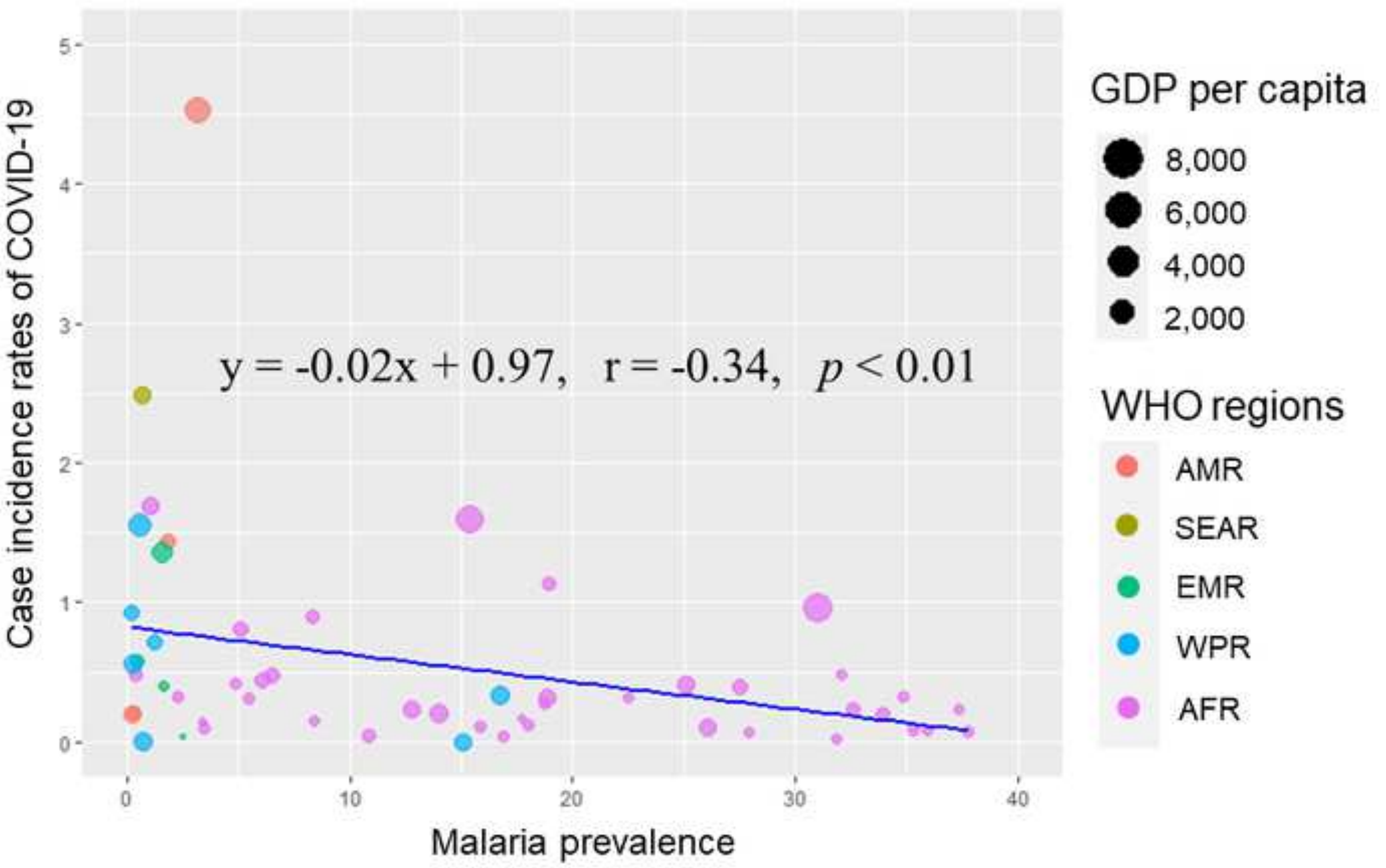

Supporting Information Figure S1

Click here to access/download
Supporting Information
Fig S1 .tif

Supporting Information Table S1

Click here to access/download
Supporting Information
Table S1.pdf

Dear Editors/reviewers,

Thank you for meticulously revising and commenting on the manuscript. We carefully revised our manuscript following the reviewers' comments.

Among the comments, there were minor ones, but since some of them were critical about the reliability of the study, we would like to start the letter with an answer to that part. The major problem suggested in the reviewers' comments was that **the biases related to COVID-19 estimates might exist as major confounders** in this study.

Our group attempted to adjust biases masking the true association between COVID-19 outcomes and parasitic disease prevalence. Therefore, by referring to previous papers, **we conceptualized the actual number of the infections (*Inf*) to be a function of factor *f* and reported cases *C*, based on test positivity rate (TPR) -** an indication of appropriate testing about disease prevalence (lines 175-195, Eq 1, 2, 3). Then the linear regression analyses were **conducted again** with the new estimated number of cases. **The updated results did not lead to different conclusions from the previous ones**. Therefore, we could develop and resubmit a more solid paper, thanks to the reviewers. In addition, a few minor errors in the paper (grammar and spacing) were modified.

Considering the problem of biases and ecological fallacy, we presented the limitation of our study in the manuscript (lines 391-406). Therefore, we introduced our study as a **hypothesis-generating study** and suggested the need for further studies at the subnational or individual level to explore hypotheses further (lines 46-47, 400-406).

Research through comparisons of the COVID-19 outcomes between countries has been steadily raised to plausibly explain why the devastating effects of COVID-19 are relatively lower in developing countries. We think our study suggested one possible explanation by adopting a reliable methodology with publicly available datasets. Further studies suggesting more definitive results can be conducted (maybe by our group) at an individual level, adopting cohort studies, case-control studies, or randomized controlled trials. In this context, we think our study provides a basis for conducting more specific research in the future.

[Reviewer 1]

I just wanted a **few minor corrections and an addition to the discussion on the PDF document**. The manuscript could be accepted after the corrections are made. I've made the corrections on the PDF document that I've loaded.

Authors' response: Following your comments on the CD-147 receptor, we modified the manuscript and added some references (lines 360-366). By specifying the role of the receptor, the content described in the original manuscript could be summarized more accurately.

Before revision (line 326)

Reports from previous studies have shown that lymphocytes produced interferons as an immune response to infection by some strains of *Plasmodium*; these interferons have a protective effect against the coronaviruses responsible for MERS, SARS, and COVID-19 [41-43].

After revision (lines 360-366)

The CD-147 receptor, expressed on several blood cells and immune cells, is a common entryway for both coronavirus and malarial *Plasmodium* to invade host cells [50]. Reports from previous studies have shown that lymphocytes produced interferons as an immune response to infection by some strains of *Plasmodium*; since the pathogens share the receptor for invasion, these interferons have a protective effect against the coronaviruses responsible for MERS, SARS, and COVID-19 [51-53]. The contribution of CD-147 blockers, Azithromycin, to preventing coronavirus infection also serves as evidence for the hypothesis [50].

Reference

50. Ulrich H, Pillat MM. CD147 as a Target for COVID-19 Treatment: Suggested Effects of Azithromycin and Stem Cell Engagement. Stem Cell Rev Rep. 2020;16(3):434-40. doi: 10.1007/s12015-020-09976-7. PubMed PMID: 32307653; PubMed Central PMCID: PMCPMC7167302.

[Reviewer 2]

In the line 115, they state that the "cytokine storm" observed in some COVID-19 patients is due to excessive Th1 responses. I suggest the reformulation of that text excerpt, since various studies have shown that the **Th17-related cytokines seem to be play pivotal roles in the "cytokine storm"** observed in the severe forms of the SARS-CoV-2 infection (Wu D, Yang XO. TH17 responses in cytokine storm of COVID-19: An emerging target of JAK2 inhibitor Fedratinib. J Microbiol Immunol Infect. 2020;53(3):368-370; Vatsalya V, Li F, Frimodig JC, et al. Therapeutic Prospects for Th-17 Cell Immune Storm Syndrome and Neurological Symptoms in COVID-19: Thiamine Efficacy and Safety, In-vitro Evidence and Pharmacokinetic Profile. Preprint. medRxiv). Please, **write the meaning of the following abbreviations when they appear for the first time in the manuscript: GDP (line 36); SSA (line 53); LMICs (line 66).**

Authors' response: Following your comments on the Th-17-related "cytokine storm" observed in the severe cases of the SARS-CoV-2 infection, we revised the manuscript and added some references (lines 116-120).

In addition, we wrote the meaning of the abbreviations when they appear for the first time in the manuscript. (lines 22, 37, 54, 68, 72, 91)

Before revision (line 114)

Some patients infected with COVID-19 experience a systemic inflammatory response, called "cytokine storm," due to excessive pro-inflammatory T-helper 1 (Th1) responses, which similarly appear in some severe manifestations of malaria [26,27]. Therefore, the resemblance of immune reactions suggests that coinfection of COVID-19 and malaria might lead to synergistic epidemics and more severe manifestations.

After revision (lines 116-120)

Some patients infected with COVID-19 experience a systemic inflammatory response, called "cytokine storm," due to excessive activation of ~~pro-inflammatory T-helper 1 (Th1)~~ T-helper 17 (Th17) [27,28], which similarly appear in some severe manifestations of malaria induced by pro-inflammatory T-helper 1 (Th1)-related immune response [26,29]. Therefore, the resemblance of immune reactions suggests that coinfection of COVID-19 and malaria might lead to synergistic epidemics and more severe manifestations [26].

Reference

26. Gutman JR, Lucchi NW, Cantey PT, Steinhardt LC, Samuels AM, Kamb ML, et al. Malaria and Parasitic Neglected Tropical Diseases: Potential Syndemics with COVID-19? Am J Trop Med Hyg. 2020;103(2):572-7. Epub 20200601. doi: 10.4269/ajtmh.20-0516. PubMed PMID: 32484155; PubMed Central PMCID: PMCPMC7410484.
27. Wu D, Yang XO. TH17 responses in cytokine storm of COVID-19: An emerging target of JAK2 inhibitor Fedratinib. J Microbiol Immunol Infect. 2020;53(3):368-70. Epub 20200311. doi: 10.1016/j.jmii.2020.03.005. PubMed PMID: 32205092; PubMed Central PMCID: PMCPMC7156211.
28. Vatsalya V, Li F, Frimodig JC, Gala KS, Srivastava S, Kong M, et al. Therapeutic Prospects for Th-17 Cell Immune Storm Syndrome and Neurological Symptoms in COVID-19: Thiamine Efficacy and Safety, In-vitro Evidence and Pharmacokinetic Profile. medRxiv. 2020. Epub 20200825. doi: 10.1101/2020.08.23.20177501. PubMed PMID: 32869036; PubMed Central PMCID: PMCPMC7457607.
29. Liao YC, Liang WG, Chen FW, Hsu JH, Yang JJ, Chang MS. IL-19 induces production of IL-6 and TNF-alpha and results in cell apoptosis through TNF-alpha. J Immunol. 2002;169(8):4288-97. doi: 10.4049/jimmunol.169.8.4288. PubMed PMID: 12370360.

[Reviewer 3]

The major problem with the analysis is that they have not accounted for the biases in Covid-19 estimates which vary by countries. The Covid-19 incidence estimates are higher in countries capable of more testing, and vice-versa. The authors themselves mention in the discussion the problem of misclassification of Covid-19 cases due to testing capacity and surveillance quality and that this varies between countries, and that they might not have adjusted for all variables in this analysis. These are real issues in any analysis regarding Covid-19 cases and their associations with other factors. In addition, there are no adequate registries for reporting deaths, cause of death, as well as excess deaths in most low income countries, which again influence the analysis. Therefore, in my opinion, **the major confounders are the biases in estimates due to variable testing capacity, true Covid-19 disease burden and excess death estimates, which the authors have not attempted to adjust in their analysis**. Unless these various biases in the calculations of the country-wise estimates are adjusted in the first place, any analysis on the association of these calculations with other factors may remain biased. At least some estimates for these factors are available, i.e., country-wise tests per million population and WHO's excess death estimates attributed to Covid-19 for different countries. The analysis must be attempted after adjusting these confounding factors before this work can be considered.

Authors' response: Thank you for giving us such an insightful comment. We found your comments immensely helpful and have revised them accordingly. Our group attempted to adjust biases masking the true association between COVID-19 outcomes and parasitic disease prevalence. However, due to uncertainties in data quality, we were only able to examine the number of reported cases as an available proxy for the real COVID-19 outcome. Therefore, by referring to previous papers, **we conceptualized the actual number of the infections (*Inf*) to be a function of factor *f* and reported cases *C*, based on test positivity rate (TPR) -** an indication of appropriate testing about disease prevalence (lines 175-195, Eq 1, 2, 3). Then the linear regression analyses were **conducted again** with the new estimated number of cases.

The standard errors in the analyses using the estimated outcome variable (*Inf*) increased and the adjusted R^2 decreased slightly. This may be because the variance of the outcome variable has increased. However, **the significance of the effects, especially the main explanatory variable, the prevalence of Malaria was maintained after adjusting the biases in COVID-19 outcomes**. Therefore, we could develop and resubmit a more solid paper.

We added contents about estimating the actual number of COVID-19 cases to the INTRODUCTION (lines 124-137) and METHODS (lines 174-195) sections. Moreover, ABSTRACT, the TABLES, the RESULTS, and the DISCUSSION section of the paper were modified following the newly calculated

results (table 3, 4, 5, lines 30-31, 262-270, 299-309, 437-440). The updated results led to similar conclusions to the previous ones. A few minor errors in the paper (grammar and spacing) were modified.

In addition, we generated a conceptual graph to understand the overall flow of research and seek ways to minimize the impact of potential biases. As described in the graph, our group attempted to adjust demographic and socioeconomic confounding factors, which may mask the true association between COVID-19 outcomes and parasitic disease prevalence. The confounders included in the graph show correlation (maybe a causal relationship or not) with the primary explanatory variable and are causally related to the main outcome variable. The problem of misclassifying reported COVID-19 cases can be caused since each country has different test capabilities and surveillance quality. Therefore, we examined the causal relationship between the prevalence of Malaria and *Inf*, an actual burden of COVID-19 estimated from the reported number of COVID-19 cases using the function of Test positivity Rate to adjust the effect of the major confounding factor, test capacity, and surveillance system. We expect that calculating and utilizing the actual number of COVID-19 cases in the analyses will aid in eliminating possible biases and precisely establishing the effect of the primary explanatory variable on the burden of COVID-19. The arrows with positive or negative signs present the direction of biases.

Conceptual graph of the final model in the study

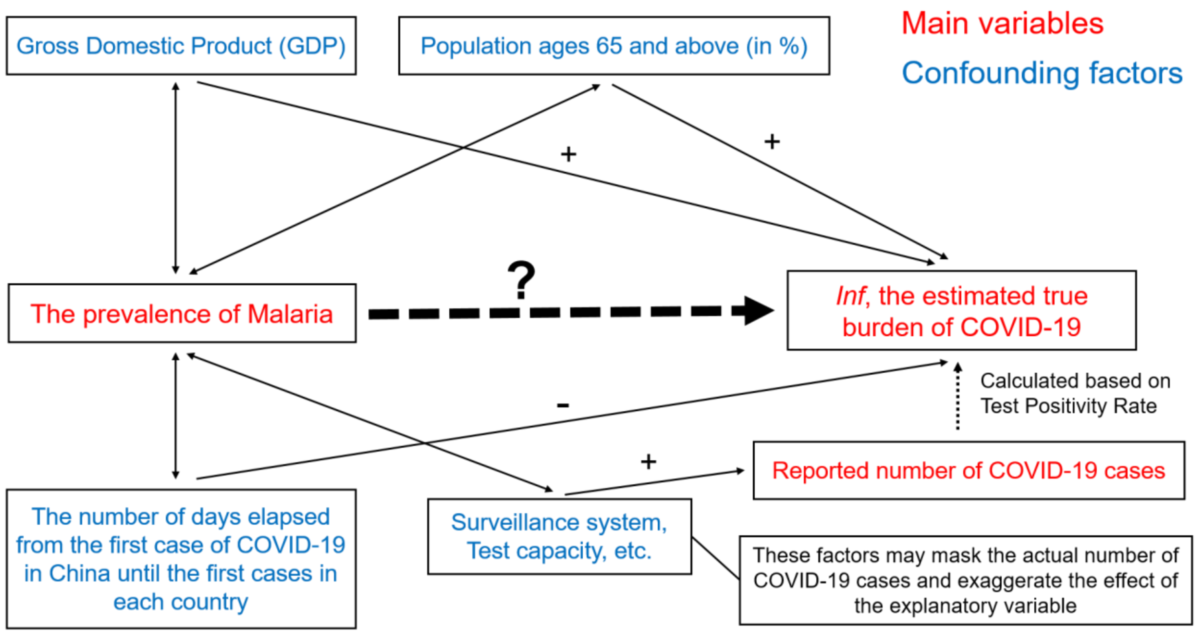

An additional paragraph in INTRODUCTION (lines 124-137)

The COVID-19 outbreak has prompted researchers to conduct nationwide comparisons to assess the effect of the pandemic. However, there are particular difficulties with interpreting comparable COVID-19 data among countries with different testing capacities, political regimes, and reporting processes [30]. Models employing transmission simulations and flight data have been used to estimate the number of undetected incidents [31,32]. These methods often need location-specific inputs, which restricts their adaptability and scalability [33]. Alternatively, the infection-fatality rate (IFR) or case-fatality rate (CFR) of COVID-19 based on serological testing and extensive diagnostic testing has been utilized to determine the actual extent of the pandemic in previous cases [34,35]. However, the IFR can be impacted by variables, including the political system, demographics, and health system capability. Furthermore, the degree to which the mortality count is accurate varies depending on how COVID-19-related fatalities are defined and how testing is conducted [33]. Researchers should employ a straightforward, comprehensive measure that is scalable across all nations and regions to enable statistical methods like multiple linear regression so they may fully evaluate the real amount of morbidity brought on by COVID-19.

Reference

31. Tuite AR, Ng V, Rees E, Fisman D. Estimation of COVID-19 outbreak size in Italy. Lancet Infect Dis. 2020;20(5):537. Epub 20200319. doi: 10.1016/S1473-3099(20)30227-9. PubMed PMID: 32199494; PubMed Central PMCID: PMCPMC7271205.
32. Giordano G, Blanchini F, Bruno R, Colaneri P, Di Filippo A, Di Matteo A, et al. Modelling the COVID-19 epidemic and implementation of population-wide interventions in Italy. Nat Med. 2020;26(6):855-60. Epub 20200422. doi: 10.1038/s41591-020-0883-7. PubMed PMID: 32322102; PubMed Central PMCID: PMCPMC7175834.
33. Kuster AC, Overgaard HJ. A novel comprehensive metric to assess effectiveness of COVID-19 testing: Inter-country comparison and association with geography, government, and policy response. PLoS One. 2021;16(3):e0248176. Epub 20210305. doi: 10.1371/journal.pone.0248176. PubMed PMID: 33667280; PubMed Central PMCID: PMCPMC7935311.
34. Fraser C, Donnelly CA, Cauchemez S, Hanage WP, Van Kerkhove MD, Hollingsworth TD, et al. Pandemic potential of a strain of influenza A (H1N1): early findings. Science. 2009;324(5934):1557-61. Epub 20090511. doi: 10.1126/science.1176062. PubMed PMID: 19433588; PubMed Central PMCID: PMCPMC3735127.
35. Nishiura H, Klinkenberg D, Roberts M, Heesterbeek JA. Early epidemiological assessment of the virulence of emerging infectious diseases: a case study of an influenza pandemic. PLoS One. 2009;4(8):e6852. Epub 20090831. doi: 10.1371/journal.pone.0006852. PubMed PMID: 19718434; PubMed Central PMCID: PMCPMC2729920.

An additional paragraph in METHODS (lines 174-195)

We evaluated the association between the disease outcome and indicators of parasitic diseases using a linear regression model framework. However, due to uncertainties in data quality, we were only able to examine the number of reported cases as an available proxy for the real COVID-19 outcome. Therefore, we conceptualized the true number of the infections (*Inf*) to be a function of factor *f* and reported cases *C* (Eq 1).

$$Inf = fC \tag{1}$$

To create a numerical value for *f*, two versions of *f* (f_1 and f_2) were considered following a previous study [33]; f_1 was obtained using CFR, while f_2 utilized TPR. The factor f_1 was suggested to effectively reflect *Inf* since CFR has been employed as a real-time way to monitor underreporting and an indication of the actual prevalence of an infectious illness in previous situations [34,35]. Nonetheless, mortality statistics may also be underreported in areas with insufficient or low testing levels, making CFR a less representative indication. Therefore, in this study, the simpler and more comprehensive factor f_2 was utilized to estimate *Inf* (Eq2), based on test positivity rate (TPR) - an indication of appropriate testing in relation to disease prevalence (Eq3). The data on the number of tests per million was collected from WDI [37].

$$f_2 = \begin{cases} 1 & , \quad if \;\; TPR < 0.05 \\ TPR/0.05 & , \quad if \;\; TPR \geq 0.05 \end{cases} \tag{2}$$

$$TPR = \frac{C}{T} \tag{3}$$

When TPR is less than 5%, the World Health Organization (WHO) recommends that testing capacity is acceptable [40]. If TPR was more than 5 percent, it was assumed that a growing number of instances were being unreported and that the factor f_2 would be equal to the TPR's ratio to that of 5 percent, the threshold (Eq2). The estimated *Inf* was used as a response variable in linear regression models in this study.

Reference

33. Kuster AC, Overgaard HJ. A novel comprehensive metric to assess effectiveness of COVID-19 testing: Inter-country comparison and association with geography, government, and policy response. PLoS One. 2021;16(3):e0248176. Epub 20210305. doi: 10.1371/journal.pone.0248176. PubMed PMID: 33667280; PubMed Central PMCID: PMCPMC7935311.
34. Fraser C, Donnelly CA, Cauchemez S, Hanage WP, Van Kerkhove MD, Hollingsworth TD, et al. Pandemic potential of a strain of influenza A (H1N1): early findings. Science. 2009;324(5934):1557-61. Epub 20090511. doi: 10.1126/science.1176062. PubMed PMID: 19433588; PubMed Central PMCID: PMCPMC3735127.
35. Nishiura H, Klinkenberg D, Roberts M, Heesterbeek JA. Early epidemiological assessment of the virulence of emerging infectious diseases: a case study of an influenza pandemic. PLoS One. 2009;4(8):e6852. Epub 20090831. doi: 10.1371/journal.pone.0006852. PubMed PMID: 19718434; PubMed Central PMCID: PMCPMC2729920.
37. World Development Indicators. Washington, USA: The World Bank; [cited 2021 October 1]. Available from: https://databank.worldbank.org/home.
40. Stein R, Wroth C, Hurt A. U.S. Coronavirus Testing Still Falls Short. How's Your State Doing?2020 [cited 2022 01 July]. Available from: https://www.npr.org/sections/health-shots/2020/05/07/851610771/u-s-coronavirus-testing-still-falls-short-hows-your-state-doing

Before revision in ABSTRACT (line 30)

A country-level ecological study using publicly available data of countries was conducted. We employed linear regression analysis to assess the association between COVID-19 and parasitic diseases. We considered demographic, socioeconomic, and geographic confounders previously suggested. A notable heterogeneity was observed across WHO regions. The countries in Africa (AFRO) showed the lowest rates of COVID-19 incidence, and the countries in the Americas (AMRO) presented the highest. The multivariable model results were computed using 165 countries, excluding missing values. In the models analyzed, lower COVID-19 incidence rates were consistently observed in malaria-endemic countries, even accounting for potential confounding variables (GDP per capita, population aged 65 and above, differences in the duration of COVID-19). However, the other parasitic diseases were not significantly associated with the spread of the pandemic.

After revision in ABSTRACT (lines 30-31)

A country-level ecological study using publicly available data of countries was conducted. **We conceptualized the true number of COVID-19 infections based on a function of test positivity rate (TPR) and employed linear regression analysis to assess the association between the outcome and parasitic diseases.** We considered demographic, socioeconomic, and geographic confounders previously suggested. A notable heterogeneity was observed across WHO regions. The countries in Africa (AFRO) showed the lowest rates of COVID-19 incidence, and the countries in the Americas (AMRO) presented the highest. The multivariable model results were computed using 165 countries,

excluding missing values. In the models analyzed, lower COVID-19 incidence rates were consistently observed in malaria-endemic countries, even accounting for potential confounding variables, Gross Domestic Product (GDP) per capita, the population aged 65 and above, and differences in the duration of COVID-19. However, the other parasitic diseases were not significantly associated with the spread of the pandemic.

Before revision in RESULTS (line 230)

A multivariable model with parasitic diseases including malaria, schistosomiasis, and STH was analyzed as a fundamental model (Table 4). COVID-19 incidence rates were significantly lower in countries endemic for malaria (β coefficient = -0.86; 95 % CI of -1.44 to -0.44; *p* < 0.01) and STH (β coefficient = -0.73; 95 % CI of -1.12 to -0.36; *p* < 0.01). The countries where schistosomiasis is prevailing, on the other hand, were not revealed to have a significant association with COVID-19 incidence rates, though the coefficient was negative (β coefficient = -0.48; 95 % CI of -1.04 to 0.07; *p* = 0.07).

After revision in RESULTS (lines 263-271)

A multivariable model with parasitic diseases including malaria, schistosomiasis, and STHs was analyzed as a fundamental model (Table 4). COVID-19 incidence rates were significantly lower in countries endemic for malaria ~~(β coefficient = -0.86; 95 % CI of -1.44 to -0.44; *p* < 0.01)~~ **(β coefficient = -1.38; 95 % CI of -2.33 to -0.41; *p* < 0.01)** ~~and STH (β coefficient = -0.73; 95 % CI of -1.12 to -0.36; *p* < 0.01)~~. The countries where schistosomiasis or STHs are prevailing, on the other hand, were not revealed to have a significant association with COVID-19 incidence rates, though the coefficients were both negative for schistosomiasis ~~(β coefficient = -0.48; 95 % CI of -1.04 to 0.07; *p* = 0.07)~~ **(β coefficient = -0.84; 95 % CI of -1.76 to 0.15; *p* = 0.09) and STHs (β coefficient = -0.58; 95 % CI of -1.20 to 0.01; *p* = 0.05).**

Before revision in RESULTS (line 259)

After screening significant variables using univariable analysis, variables with pairwise correlation coefficients between -0.7 and 0.7 were comprised for further multivariable linear regression (S1 Fig). The saturated model had the least value of AIC and BIC (S1 Table). Since the AIC and BIC significantly increased when the parasitic diseases variables were removed from the model, it could be concluded that the variables explain a considerable level of variation in the saturated model. A total of 165

countries were retained in the model, excluding missing values. Table 5 summarizes key results from the multivariable regression model of imputed predictive variables for the response variable, COVID-19 incidence rates. In the model, the malaria-endemic countries presented significantly lower COVID-19 incidence rates (β coefficient = -0.40; 95 % CI of -0.79 to -0.06; *p* < 0.05) even adjusting for confounding variables, the timing of the disease eruption (duration variable), the proportion of the population over 65, and GDP per capita, while schistosomiasis (β coefficient = -0.19; 95 % CI of -0.58 to 0.18; *p* = 0.31) and STH (β coefficient = -0.18; 95 % CI of -0.46 to 0.10; *p* = 0.21) were not significantly associated with the response variable. In addition, the model examined GDP per capita and the timing of the disease eruption to have significant associations for COVID-19 incidence rates. In the univariable regression model, including a predictor, the prevalence of malaria, and an outcome, COVID-19 incidence rates, the outcome variable monotonically decreased as the predictor increased (β coefficient = -0.02; 95 % CI of -0.04 to -0.01; *p* < 0.01) (Fig 3).

After revision in RESULTS (lines 292-310)

After screening significant variables using univariable analysis, variables with pairwise correlation coefficients between -0.7 and 0.7 were comprised for further multivariable linear regression (S1 Fig). The saturated model had the least value of AIC and BIC (S1 Table). Since the AIC and BIC significantly increased when the parasitic disease variables were removed from the model, it could be concluded that the variables explain a considerable level of variation in the saturated model. A total of 165 countries were retained in the model, excluding missing values. Table 5 summarizes key results from the multivariable regression model of imputed predictive variables for the response variable, COVID-19 incidence rates. In the model, the malaria-endemic countries presented significantly lower COVID-19 incidence rates ~~(β coefficient = -0.40; 95 % CI of -0.79 to -0.06; *p* < 0.05)~~ **(β coefficient = -0.73; 95 % CI of -1.46 to -0.10; *p* < 0.05)** even adjusting for confounding variables, the timing of the disease eruption (duration variable), the proportion of the population over 65, and GDP per capita, while schistosomiasis ~~(β coefficient = -0.19; 95 % CI of -0.58 to 0.18; *p* = 0.31)~~ **(β coefficient = -0.30; 95 % CI of -1.02 to 0.40; *p* = 0.39) and STHs** ~~(β coefficient = -0.18; 95 % CI of -0.46 to 0.10; *p* = 0.21)~~ **(β coefficient = -0.14; 95 % CI of -0.67 to 0.39; *p* = 0.59)** were not significantly associated with the response variable. In addition, the model examined GDP per capita ~~and the timing of the disease eruption~~ to have significant associations for COVID-19 incidence rates. In the univariable regression model ~~including a predictor,~~ with the prevalence of malaria as a predictor, and COVID-19 incidence rates as an outcome, the outcome variable monotonically decreased as the predictor increased **(β coefficient = -0.02; 95 % CI of -0.04 to -0.01; *p* < 0.01)** (Fig 3).

Before revision in DISCUSSION (line 398)

Due to the differences in health infrastructure and testing policy, the data may obscure the accurate size of the diseases [5]. Studies have revealed significantly under-reported cases and associated deaths in several countries, especially in low-income countries [55]. Thus, our results should be cautiously interpreted. We also want to point out that we might not have adjusted for all confounding variables, though we attempted to include demographic, socioeconomic, and geographic variables suggested in previous studies. Our study is based on an analysis describing a still evolving and imperfectly explored epidemic, only considering the scientific understanding of the disease at the time. Therefore, due to these unknown factors, our study could not deal with all factors that influence the spread of COVID-19 and residual confounding.

After revision in DISCUSSION (lines 435-445)

Due to the differences in health infrastructure and testing policy, the data may obscure the accurate size of the diseases [5]. Studies have revealed significantly under-reported cases and associated deaths in several countries, especially in low-income countries [65]. **Even though we conceptualized the true number of the infections based on TPR to adjust possible biases in the under-reporting of the COVID-19 cases, we cannot conclude that our study has perfectly precluded them.** Thus, our results should be cautiously interpreted. We also want to point out that we might not have adjusted for all confounding variables, though we attempted to include demographic, socioeconomic, and geographic variables suggested in previous studies. Our study is based on an analysis describing a still evolving and imperfectly explored epidemic, only considering the scientific understanding of the disease at the time. Therefore, due to these unknown factors, our study could not deal with all factors that influence the spread of COVID-19 and residual confounding.

Table 3. Univariable analyses of predictive variables and root-transformed COVID-19 incidence rates.

Variable	Number of countries examined (%)[1]	Coefficient (95% CI)	SE of coefficient	Coefficient (95% CI) /bootstrapping	SE of coefficient /bootstrapping	*p*-value of coefficient[2]	ΔR^2
**Malaria (in %)**							
Non-endemic	156 (78.79)	Reference		Reference			
Endemic	42 (21.21)	~~-1.59 (-1.96, -1.23)~~	~~0.18~~	~~-1.59 (-1.82, -1.36)~~	~~0.12~~	~~< 0.001~~	~~0.28~~
		-2.29 (-2.89, -1.69)	0.30	-2.29 (-2.64, -1.86)	0.19	< 0.001	0.23
**Schistosomiasis (in %)**							
Non-endemic	157 (77.73)	Reference		Reference			
Endemic	45 (22.27)	~~-1.53 (-1.89, -1.17)~~	~~0.18~~	~~-1.53 (-1.75, -1.24)~~	~~0.13~~	~~< 0.001~~	~~0.26~~
		-2.19 (-2.82, -1.56)	0.31	-2.19 (-2.63, -1.75)	0.30	< 0.001	0.19
**STHs (in %)**							
Non-endemic	146 (73.74)	Reference		Reference			
Endemic	52 (26.26)	~~-1.28 (-1.63, -0.93)~~	~~0.18~~	~~-1.28 (-1.56, -0.98)~~	~~0.14~~	~~< 0.001~~	~~0.2~~
		-1.49 (-2.13, -0.85)	0.32	-1.49 (-2.06, -0.86)	0.30	< 0.001	0.11
**Tuberculosis (in %)**							
Low (< 0.01)	55 (29.41)	Reference		Reference			
Moderate (< 0.1)	91 (48.66)	~~-0.97 (-1.33, -0.61)~~	~~0.18~~	~~-0.97 (-1.33, -0.61)~~	~~0.19~~	~~< 0.001~~	~~0.27~~
		-0.99 (-1.63, -0.35)	0.32	-0.99 (-1.77, -0.61)	0.36	< 0.001	0.17
High (0.1 ≤)	41 (21.93)	~~-1.82 (-2.26, -1.39)~~	~~0.22~~	~~-1.82 (-2.19, -1.46)~~	~~0.19~~	~~< 0.001~~	

		-2.38 (-3.16, -1.61)	0.39	-2.38 (-3.11, -1.72)	0.34	< 0.001	
**Population ages 65 and above (in %)**							
< 5	72 (38.30)	Reference		Reference			
< 15	70 (37.23)	~~1.13 (0.81, 1.45)~~	~~0.16~~	~~1.13 (0.77, 1.46)~~	~~0.16~~	~~< 0.001~~	~~0.37~~
		2.15 (1.54, 2.75)	0.35	2.15 (1.54, 2.80)	0.34	< 0.001	0.26
15 ≤	46 (24.47)	~~1.87 (1.51, 2.24)~~	~~0.18~~	~~1.87 (1.51, 2.19)~~	~~0.16~~	~~< 0.001~~	
		2.28 (1.60, 2.97)	0.32	2.28 (1.74, 2.88)	0.28	< 0.001	
**Death by non-communicable diseases (in %)**							
< 60	50 (27.17)	Reference		Reference			
< 80	54 (29.35)	~~1.04 (0.67, 1.41)~~	~~0.18~~	~~1.04 (0.71, 1.38)~~	~~0.17~~	~~< 0.001~~	~~0.41~~
		1.96 (1.26, 2.65)	0.35	1.96 (1.32, 2.70)	0.17	< 0.001	0.25
80 ≤	80 (43.48)	~~1.92 (1.57, 2.25)~~	~~0.17~~	~~1.92 (1.61, 2.18)~~	~~0.14~~	~~< 0.001~~	
		2.51 (1.87, 3.15)	0.32	2.51 (2.03, 2.96)	0.14	< 0.001	
**Diabetes (in %)**							
< 5	39 (19.31)	Reference		Reference			
< 10	103 (50.99)	~~0.68 (0.23, 1.14)~~	~~0.23~~	~~0.68 (0.18, 1.02)~~	~~0.23~~	~~< 0.01~~	~~0.04~~
		1.17 (0.41, 1.93)	0.38	1.17 (0.53, 1.78)	0.31	< 0.01	0.05
10 ≤	60 (29.70)	~~0.55 (0.05, 1.04)~~	~~0.25~~	~~0.55 (0.05, 1.02)~~	~~0.25~~	~~0.03~~	
		1.13 (0.29, 1.96)	0.41	1.13 (0.38, 1.98)	0.40	< 0.01	
**GDP per capita (in $)**							

< 2000	48 (24.87)	Reference		Reference			
< 6,000	43 (22.28)	~~0.96 (0.58, 1.35)~~	~~0.19~~	~~0.96 (0.63, 1.33)~~	~~0.16~~	~~< 0.001~~	~~0.44~~
		1.70 (0.99, 2.43)	0.36	1.70 (1.13, 2.36)	0.30	< 0.001	0.31
< 18,000	53 (27.46)	~~1.83 (1.46, 2.19)~~	~~0.19~~	~~1.83 (1.54, 2.14)~~	~~0.15~~	~~< 0.001~~	
		3.02 (2.33, 3.71)	0.34	3.02 (2.41, 3.73)	0.33	< 0.001	
18,000 ≤	49 (25.39)	~~2.13 (1.76, 2.50)~~	~~0.19~~	~~2.13 (1.83, 2.43)~~	~~0.15~~	~~< 0.001~~	
		2.26 (1.56, 2.96)	0.35	2.26 (1.86, 2.69)	0.21	< 0.001	
**Current health expenditure (% GDP)**							
< 5	60 (38.08)	Reference		Reference			
< 8	81 (43.32)	~~0.84 (0.45, 1.24)~~	~~0.19~~	~~0.84 (0.47, 1.21)~~	~~0.19~~	~~< 0.001~~	~~0.13~~
		1.59 (0.94, 2.24)	0.32	1.59 (0.94, 2.24)	0.27	< 0.001	0.14
8 ≤	46 (24.60)	~~1.11 (0.65, 1.55)~~	~~0.23~~	~~1.11 (0.64, 1.53)~~	~~0.22~~	~~< 0.001~~	
		1.74 (1.01, 2.49)	0.37	1.74 (1.01, 2.49)	0.35	< 0.001	
**Average surface temperature (in ℃)**							
Polar-Microthermal (< 18)	66 (33.67)	Reference		Reference			
Temperate (< 26)	60 (30.61)	~~-1.25 (-1.64, -0.86)~~	~~0.20~~	~~-1.25 (-1.61, -0.89)~~	~~0.18~~	~~< 0.001~~	~~0.2~~
		-1.25 (-1.93, -0.51)	0.36	-1.25 (-1.85, -0.55)	0.33	< 0.001	0.11
Tropical (≤ 26)	70 (35.71)	~~-1.10 (-1.48, -0.73)~~	~~0.19~~	~~-1.10 (-1.44, -0.68)~~	~~0.20~~	~~< 0.001~~	
		-1.27 (-1.96, -0.58)	0.34	-1.27 (-1.88, -0.44)	0.36	< 0.001	
**Urban population (in %)**							

< 50	67 (33.17)	Reference		Reference			
< 75	72 (35.64)	~~1.03 (0.66, 1.41)~~	~~0.19~~	~~1.03 (0.63, 1.67)~~	~~0.19~~	~~< 0.001~~	~~0.21~~
		1.91 (1.27, 2.56)	0.32	1.91 (1.33, 2.60)	0.32	< 0.001	0.17
75 ≤	63 (31.19)	~~1.33 (0.94, 1.72)~~	~~0.20~~	~~1.33 (0.96, 1.67)~~	~~0.18~~	~~< 0.001~~	
		1.69 (1.02, 2.35)	0.33	1.69 (1.15, 2.24)	0.27	< 0.001	
**Fully vaccinated with COVID-vaccine (in %)**							
< 30	86 (43.21)	Reference		Reference			
< 60	61 (30.65)	~~1.36 (1.02, 1.71)~~	~~0.17~~	~~1.36 (1.02, 1.69)~~	~~0.17~~	~~< 0.001~~	~~0.31~~
		2.20 (1.58, 2.82)	0.31	2.20 (1.55, 2.82)	0.32	< 0.001	0.30
60 ≤	52 (26.13)	~~1.42 (1.06, 1.79)~~	~~0.18~~	~~1.42 (1.05, 1.80)~~	~~0.19~~	~~< 0.001~~	
		1.27 (0.67, 1.92)	0.33	1.27 (0.71, 2.11)	0.33	< 0.001	
**Population density (per km²)**							
< 50	67 (33.83)	Reference		Reference			
< 120	59 (29.80)	~~0.21 (-0.23, 0.65)~~	~~0.22~~	~~0.21 (-0.23, 0.63)~~	~~0.22~~	~~0.34~~	~~0.02~~
		0.36 (-0.37, 1.65)	0.37	0.36 (-0.36, 1.45)	0.34	0.34	0.02
120 ≤	72 (36.36)	~~0.37 (-0.05, 0.78)~~	~~0.21~~	~~0.37 (-0.03, 0.78)~~	~~0.21~~	~~0.08~~	
		0.20 (-0.50, 0.98)	0.35	0.20 (-0.47, 0.88)	0.36	0.57	
**Human development index**							
< 0.6	46 (24.60)	Reference		Reference			
< 0.8	76 (40.64)	~~1.32 (1.01, 1.64)~~	~~0.16~~	~~1.32 (1.08, 1.56)~~	~~0.13~~	~~< 0.001~~	~~0.52~~

		2.43 (1.80, 3.06)	0.32	2.43 (1.93, 2.97)	0.32	< 0.001	0.31
0.8 ≤	65 (34.76)	~~2.33 (2.01, 2.66)~~	~~0.17~~	~~2.33 (2.07, 2.57)~~	~~0.12~~	~~< 0.001~~	
		2.85 (2.20, 3.51)	0.33	2.85 (2.48, 3.33)	0.33	< 0.001	
**Duration (in days)**							
< 70	104 (52.26)	Reference		Reference			
70 ≤	95 (47.74)	~~-1.10 (-1.41, -0.79)~~	~~0.16~~	~~-1.10 (-1.39, -0.77)~~	~~0.15~~	~~< 0.001~~	~~0.19~~
		-1.34 (-1.89, -0.79)	0.28	-1.34 (-1.89, -0.78)	0.27	< 0.001	0.19
**Mean years of schooling (in years)**							
< 7	58 (31.35)	Reference		Reference			
< 10	50 (27.03)	~~1.40 (1.04, 1.77)~~	~~0.18~~	~~1.40 (1.09, 1.71)~~	~~0.13~~	~~< 0.001~~	~~0.41~~
		1.59 (1.04, 1.77)	0.33	1.59 (1.11, 1.67)	0.28	< 0.001	0.30
10 ≤	77 (41.62)	~~1.87 (1.54, 2.20)~~	~~0.17~~	~~1.87 (1.58, 2.15)~~	~~0.12~~	~~< 0.001~~	
		1.97 (1.54, 2.20)	0.29	1.97 (1.64, 2.19)	0.31	< 0.001	

[1]Percentages reflect cases without missing values

[2]The level of statistical significance is 0.05

Table 4. Summary of associations between parasitic disease variables and root-transformed COVID-19 incidence rates. (changed)

Variable[1]	Coefficient (95% CI)	SE of coefficient	Coefficient (95% CI) /bootstrapping	SE of coefficient /bootstrapping	*p*-value of coefficient	Adjusted ΔR^2	*p*-value of ΔR^2
**Malaria (in %)**							
Non-endemic	Reference		Reference				
Endemic	~~-0.86 (-1.44, -0.44)~~<br>-1.38 (-2.33, -0.41)	~~0.28~~<br>0.48	~~-0.86 (-1.37, -0.40)~~<br>-1.38 (-2.06, -0.63)	~~0.25~~<br>0.36	~~< 0.01~~<br>< 0.01		
**Schistosomiasis (in %)**							
Non-endemic	Reference		Reference			0.24	< 0.001
Endemic	~~-0.48 (-1.04, 0.07)~~<br>-0.84 (-1.76, 0.15)	~~0.29~~<br>0.48	~~-0.48 (-0.98, 0.00)~~<br>-0.84 (-1.57, -0.04)	~~0.26~~<br>0.38	~~0.07~~<br>0.09		
**STHs (in %)**							
Non-endemic	Reference		Reference				
Endemic	~~-0.73 (-1.12, -0.36)~~<br>-0.58 (-1.20, 0.01)	~~0.19~~<br>0.32	~~-0.73 (-1.04, -0.45)~~<br>-0.58 (-1.15, 0.02)	~~0.16~~<br>0.32	~~< 0.01~~<br>0.05		

[1]Interaction terms were not significant

Table 5. Summary of multivariable linear regression, including parasite variables of interest and potential confounders. (changed)

**Variable**	**Coefficient (95% CI)**	**SE of coefficient**	**Coefficient (95% CI) /bootstrapping**	**SE of coefficient /bootstrapping**	***p*-value of coefficient**	**Adjusted ΔR^2**	***p*-value of ΔR^2**
**Malaria**							
Non-endemic	Reference		Reference				
Endemic	~~-0.40 (-0.79, -0.06)~~<br>-0.73 (-1.46, -0.10)	~~0.2~~<br>0.36	~~-0.40 (-0.83, -0.12)~~<br>-0.73 (-1.42, -0.12)	~~0.18~~<br>0.34	~~< 0.05~~<br>< 0.05		
**Schistosomiasis**							
Non-endemic	Reference		Reference				
Endemic	~~-0.19 (-0.58, 0.18)~~<br>-0.30 (-1.02, 0.40)	~~0.2~~<br>0.36	~~-0.19 (-0.48, 0.05)~~<br>-0.30 (-1.06, 0.34)	~~0.19~~<br>0.36	~~0.31~~<br>0.39		
**STHs**							
Non-endemic	Reference		Reference				
Endemic	~~-0.18 (-0.46, 0.10)~~<br>-0.14 (-0.67, 0.39)	~~0.15~~<br>0.26	~~-0.18 (-0.48, 0.01)~~<br>-0.14 (-0.64, 0.45)	~~0.12~~<br>0.28	~~0.21~~<br>0.59		
**Duration (in days)**							
< 70	Reference		Reference			~~0.69~~<br>0.49	~~< 0.001~~<br>< 0.001
70 ≤	~~-0.36 (-0.61, -0.10)~~<br>-0.40 (-0.87, 0.07)	~~0.12~~<br>0.24	~~-0.36 (-0.41, -0.12)~~<br>-0.40 (-0.88, 0.09)	~~0.11~~<br>0.29	~~< 0.01~~<br>0.11		
**Population ages 65 and above (in %)**							
< 5	Reference		Reference				
< 15	~~-0.06 (-0.34, 0.28)~~	~~0.16~~	~~-0.06 (-0.40, 0.35)~~	~~0.19~~	~~0.86~~		

	0.17(-0.41, 0.74)	0.29	0.17(-0.45, 0.80)	0.33	0.56
15 ≤	~~0.23 (-0.13, 0.63)~~	~~0.19~~	~~0.23 (-0.17, 0.72)~~	~~0.22~~	~~0.20~~
	0.35 (-0.38, 1.04)	0.35	0.35 (-0.35, 1.02)	0.36	0.36
**GDP per capita (in $)**					
< 2000	Reference		Reference		
< 6,000	~~0.43 (0.10, 0.77)~~	~~0.15~~	~~0.43 (0.15, 0.69)~~	~~0.15~~	~~< 0.05~~
	0.88 (0.25, 1.51)	0.31	0.88 (0.26, 1.58)	0.32	< 0.001
< 18,000	~~1.3 (0.94, 1.72)~~	~~0.18~~	~~1.3 (0.94, 1.61)~~	~~0.21~~	~~< 0.001~~
	1.86 (1.12, 2.59)	0.37	1.86 (1.26, 2.64)	0.34	< 0.001
18,000 ≤	~~0.95 (0.95, 1.87)~~	~~0.19~~	~~0.95 (0.98, 1.52)~~	~~0.22~~	~~< 0.001~~
	0.96 (0.12, 1.80)	0.41	0.96 (0.22, 1.78)	0.39	< 0.05

Click here to access/download
Revised Article with Changes Highlighted
COVID-19-parasite-manuscript_revision_edit_rev1.docx
