# Supplementary material for: The notable global heterogeneity in the distribution of COVID-19 cases and the association with pre-existing parasitic diseases
Source: PLoS Negl Trop Dis. 2022 Oct 10;16(10):e0010826. doi: 10.1371/journal.pntd.0010826 (PMC9584393; doi:10.1371/journal.pntd.0010826)
Supplement: Attachment — Submitted filename: A letter to reviewers_rev1.docx [file pntd.0010826.s004.docx]

Dear Editors/reviewers,

Thank you for meticulously revising and commenting on the manuscript. We carefully revised our manuscript following the reviewers’ comments.

Among the comments, there were minor ones, but since some of them were critical about the reliability of the study, we would like to start the letter with an answer to that part. The major problem suggested in the reviewers’ comments was that **the biases related to COVID-19 estimates might exist as major confounders** in this study.

Our group attempted to adjust biases masking the true association between COVID-19 outcomes and parasitic disease prevalence. Therefore, by referring to previous papers, **we conceptualized the actual number of the infections (*Inf*) to be a function of factor *f* and reported cases *C*, based on test positivity rate (TPR) -** an indication of appropriate testing about disease prevalence (lines 175-195, Eq 1, 2, 3). Then the linear regression analyses were **conducted again** with the new estimated number of cases. **The updated results did not lead to different conclusions from the previous ones**. Therefore, we could develop and resubmit a more solid paper, thanks to the reviewers. In addition, a few minor errors in the paper (grammar and spacing) were modified.

Considering the problem of biases and ecological fallacy, we presented the limitation of our study in the manuscript (lines 391-406). Therefore, we introduced our study as a **hypothesis-generating study** and suggested the need for further studies at the subnational or individual level to explore hypotheses further (lines 46-47, 400-406).

Research through comparisons of the COVID-19 outcomes between countries has been steadily raised to plausibly explain why the devastating effects of COVID-19 are relatively lower in developing countries. We think our study suggested one possible explanation by adopting a reliable methodology with publicly available datasets. Further studies suggesting more definitive results can be conducted (maybe by our group) at an individual level, adopting cohort studies, case-control studies, or randomized controlled trials. In this context, we think our study provides a basis for conducting more specific research in the future.

**[Reviewer 1]**

I just wanted a **few minor corrections and an addition to th**e **discussion on the PDF document**. The manuscript could be accepted after the corrections are made. I've made the corrections on the PDF document that I've loaded.

**Authors’ response:** Following your comments on the CD-147 receptor, we modified the manuscript and added some references (lines 360-366). By specifying the role of the receptor, the content described in the original manuscript could be summarized more accurately.

**Before revision (line 326)**

Reports from previous studies have shown that lymphocytes produced interferons as an immune response to infection by some strains of *Plasmodium*; these interferons have a protective effect against the coronaviruses responsible for MERS, SARS, and COVID-19 [41-43].

**After revision (lines 360-366)**

The CD-147 receptor, expressed on several blood cells and immune cells, is a common entryway for both coronavirus and malarial *Plasmodium* to invade host cells [50]. Reports from previous studies have shown that lymphocytes produced interferons as an immune response to infection by some strains of *Plasmodium*; since the pathogens share the receptor for invasion, these interferons have a protective effect against the coronaviruses responsible for MERS, SARS, and COVID-19 [51-53]. The contribution of CD-147 blockers, Azithromycin, to preventing coronavirus infection also serves as evidence for the hypothesis [50].

**Reference**

50. Ulrich H, Pillat MM. CD147 as a Target for COVID-19 Treatment: Suggested Effects of Azithromycin and Stem Cell Engagement. Stem Cell Rev Rep. 2020;16(3):434-40. doi: 10.1007/s12015-020-09976-7. PubMed PMID: 32307653; PubMed Central PMCID: PMCPMC7167302.

**[Reviewer 2]**

**In the line 115,** they state that the “cytokine storm” observed in some COVID-19 patients is due to excessive Th1 responses. I suggest the reformulation of that text excerpt, since various studies have shown that the **Th17-related cytokines seem to be play pivotal roles in the “cytokine storm”** observed in the severe forms of the SARS-CoV-2 infection (Wu D, Yang XO. TH17 responses in cytokine storm of COVID-19: An emerging target of JAK2 inhibitor Fedratinib. J Microbiol Immunol Infect. 2020;53(3):368-370; Vatsalya V, Li F, Frimodig JC, et al. Therapeutic Prospects for Th-17 Cell Immune Storm Syndrome and Neurological Symptoms in COVID-19: Thiamine Efficacy and Safety, In-vitro Evidence and Pharmacokinetic Profile. Preprint. medRxiv). Please, **write the meaning of the following abbreviations when they appear for the first time in the manuscript: GDP (line 36); SSA (line 53); LMICs (line 66).**

**Authors’ response:** Following your comments on the Th-17-related “cytokine storm” observed in the severe cases of the SARS-CoV-2 infection, we revised the manuscript and added some references (lines 116-120).

In addition, we wrote the meaning of the abbreviations when they appear for the first time in the manuscript. (lines 22, 37, 54, 68, 72, 91)

**Before revision (line 114)**

Some patients infected with COVID-19 experience a systemic inflammatory response, called “cytokine storm,” due to excessive pro-inflammatory T-helper 1 (Th1) responses, which similarly appear in some severe manifestations of malaria [26,27]. Therefore, the resemblance of immune reactions suggests that coinfection of COVID-19 and malaria might lead to synergistic epidemics and more severe manifestations.

**After revision (lines 116-120)**

Some patients infected with COVID-19 experience a systemic inflammatory response, called “cytokine storm,” due to excessive activation of ~~pro-inflammatory T-helper 1 (Th1)~~ T-helper 17 (Th17) [27,28], which similarly appear in some severe manifestations of malaria induced by pro-inflammatory T-helper 1 (Th1)-related immune response [26,29]. Therefore, the resemblance of immune reactions suggests that coinfection of COVID-19 and malaria might lead to synergistic epidemics and more severe manifestations [26].

**Reference**

26. Gutman JR, Lucchi NW, Cantey PT, Steinhardt LC, Samuels AM, Kamb ML, et al. Malaria and Parasitic Neglected Tropical Diseases: Potential Syndemics with COVID-19? Am J Trop Med Hyg. 2020;103(2):572-7. Epub 20200601. doi: 10.4269/ajtmh.20-0516. PubMed PMID: 32484155; PubMed Central PMCID: PMCPMC7410484.

27. Wu D, Yang XO. TH17 responses in cytokine storm of COVID-19: An emerging target of JAK2 inhibitor Fedratinib. J Microbiol Immunol Infect. 2020;53(3):368-70. Epub 20200311. doi: 10.1016/j.jmii.2020.03.005. PubMed PMID: 32205092; PubMed Central PMCID: PMCPMC7156211.

28. Vatsalya V, Li F, Frimodig JC, Gala KS, Srivastava S, Kong M, et al. Therapeutic Prospects for Th-17 Cell Immune Storm Syndrome and Neurological Symptoms in COVID-19: Thiamine Efficacy and Safety, In-vitro Evidence and Pharmacokinetic Profile. medRxiv. 2020. Epub 20200825. doi: 10.1101/2020.08.23.20177501. PubMed PMID: 32869036; PubMed Central PMCID: PMCPMC7457607.

29. Liao YC, Liang WG, Chen FW, Hsu JH, Yang JJ, Chang MS. IL-19 induces production of IL-6 and TNF-alpha and results in cell apoptosis through TNF-alpha. J Immunol. 2002;169(8):4288-97. doi: 10.4049/jimmunol.169.8.4288. PubMed PMID: 12370360.

**[Reviewer 3]**

**The major problem with the analysis is that they have not accounted for the biases in Covid-19 estimates which vary by countries**. The Covid-19 incidence estimates are higher in countries capable of more testing, and vice-versa. The authors themselves mention in the discussion the problem of misclassification of Covid-19 cases due to testing capacity and surveillance quality and that this varies between countries, and that they might not have adjusted for all variables in this analysis. These are real issues in any analysis regarding Covid-19 cases and their associations with other factors. In addition, there are no adequate registries for reporting deaths, cause of death, as well as excess deaths in most low income countries, which again influence the analysis. Therefore, in my opinion, **the major confounders are the biases in estimates due to variable testing capacity, true Covid-19 disease burden and excess death estimates, which the authors have not attempted to adjust in their analysis**. Unless these various biases in the calculations of the country-wise estimates are adjusted in the first place, any analysis on the association of these calculations with other factors may remain biased. At least some estimates for these factors are available, i.e., country-wise tests per million population and WHO's excess death estimates attributed to Covid-19 for different countries. The analysis must be attempted after adjusting these confounding factors before this work can be considered.

**Authors’ response:** Thank you for giving us such an insightful comment. We found your comments immensely helpful and have revised them accordingly. Our group attempted to adjust biases masking the true association between COVID-19 outcomes and parasitic disease prevalence. However, due to uncertainties in data quality, we were only able to examine the number of reported cases as an available proxy for the real COVID-19 outcome. Therefore, by referring to previous papers, **we conceptualized the actual number of the infections (*Inf*) to be a function of factor *f* and reported cases *C*, based on test positivity rate (TPR) -** an indication of appropriate testing about disease prevalence (lines 175-195, Eq 1, 2, 3). Then the linear regression analyses were **conducted again** with the new estimated number of cases.

The standard errors in the analyses using the estimated outcome variable (*Inf*) increased and the adjusted R^2^ decreased slightly. This may be because the variance of the outcome variable has increased. However, **the significance of the effects, especially the main explanatory variable, the prevalence of Malaria was maintained after adjusting the biases in COVID-19 outcomes**. Therefore, we could develop and resubmit a more solid paper.

We added contents about estimating the actual number of COVID-19 cases to the INTRODUCTION (lines 124-137) and METHODS (lines 174-195) sections. Moreover, ABSTRACT, the TABLES, the RESULTS, and the DISCUSSION section of the paper were modified following the newly calculated results (table 3, 4, 5, lines 30-31, 262-270, 299-309, 437-440). The updated results led to similar conclusions to the previous ones. A few minor errors in the paper (grammar and spacing) were modified.

In addition, we generated a conceptual graph to understand the overall flow of research and seek ways to minimize the impact of potential biases. As described in the graph, our group attempted to adjust demographic and socioeconomic confounding factors, which may mask the true association between COVID-19 outcomes and parasitic disease prevalence. The confounders included in the graph show correlation (maybe a causal relationship or not) with the primary explanatory variable and are causally related to the main outcome variable. The problem of misclassifying reported COVID-19 cases can be caused since each country has different test capabilities and surveillance quality. Therefore, we examined the causal relationship between the prevalence of Malaria and *Inf*, an actual burden of COVID-19 estimated from the reported number of COVID-19 cases using the function of Test positivity Rate to adjust the effect of the major confounding factor, test capacity, and surveillance system. We expect that calculating and utilizing the actual number of COVID-19 cases in the analyses will aid in eliminating possible biases and precisely establishing the effect of the primary explanatory variable on the burden of COVID-19. The arrows with positive or negative signs present the direction of biases.

**An additional paragraph in INTRODUCTION (lines 124-137)**

The COVID-19 outbreak has prompted researchers to conduct nationwide comparisons to assess the effect of the pandemic. However, there are particular difficulties with interpreting comparable COVID-19 data among countries with different testing capacities, political regimes, and reporting processes [30]. Models employing transmission simulations and flight data have been used to estimate the number of undetected incidents [31,32]. These methods often need location-specific inputs, which restricts their adaptability and scalability [33]. Alternatively, the infection-fatality rate (IFR) or case-fatality rate (CFR) of COVID-19 based on serological testing and extensive diagnostic testing has been utilized to determine the actual extent of the pandemic in previous cases [34,35]. However, the IFR can be impacted by variables, including the political system, demographics, and health system capability. Furthermore, the degree to which the mortality count is accurate varies depending on how COVID-19-related fatalities are defined and how testing is conducted [33]. Researchers should employ a straightforward, comprehensive measure that is scalable across all nations and regions to enable statistical methods like multiple linear regression so they may fully evaluate the real amount of morbidity brought on by COVID-19.

**Reference**

31. Tuite AR, Ng V, Rees E, Fisman D. Estimation of COVID-19 outbreak size in Italy. Lancet Infect Dis. 2020;20(5):537. Epub 20200319. doi: 10.1016/S1473-3099(20)30227-9. PubMed PMID: 32199494; PubMed Central PMCID: PMCPMC7271205.

32. Giordano G, Blanchini F, Bruno R, Colaneri P, Di Filippo A, Di Matteo A, et al. Modelling the COVID-19 epidemic and implementation of population-wide interventions in Italy. Nat Med. 2020;26(6):855-60. Epub 20200422. doi: 10.1038/s41591-020-0883-7. PubMed PMID: 32322102; PubMed Central PMCID: PMCPMC7175834.

33. Kuster AC, Overgaard HJ. A novel comprehensive metric to assess effectiveness of COVID-19 testing: Inter-country comparison and association with geography, government, and policy response. PLoS One. 2021;16(3):e0248176. Epub 20210305. doi: 10.1371/journal.pone.0248176. PubMed PMID: 33667280; PubMed Central PMCID: PMCPMC7935311.

34. Fraser C, Donnelly CA, Cauchemez S, Hanage WP, Van Kerkhove MD, Hollingsworth TD, et al. Pandemic potential of a strain of influenza A (H1N1): early findings. Science. 2009;324(5934):1557-61. Epub 20090511. doi: 10.1126/science.1176062. PubMed PMID: 19433588; PubMed Central PMCID: PMCPMC3735127.

35. Nishiura H, Klinkenberg D, Roberts M, Heesterbeek JA. Early epidemiological assessment of the virulence of emerging infectious diseases: a case study of an influenza pandemic. PLoS One. 2009;4(8):e6852. Epub 20090831. doi: 10.1371/journal.pone.0006852. PubMed PMID: 19718434; PubMed Central PMCID: PMCPMC2729920.

**An additional paragraph in METHODS (lines 174-195)**

We evaluated the association between the disease outcome and indicators of parasitic diseases using a linear regression model framework. However, due to uncertainties in data quality, we were only able to examine the number of reported cases as an available proxy for the real COVID-19 outcome. Therefore, we conceptualized the true number of the infections (*Inf*) to be a function of factor *f* and reported cases *C* (Eq 1).

$$Inf=fC$$

(1)

To create a numerical value for *f*, two versions of *f* (*f*_1_ and *f*_2_) were considered following a previous study [33]; *f*_1_ was obtained using CFR, while *f*_2_ utilized TPR. The factor f_1_ was suggested to effectively reflect *Inf* since CFR has been employed as a real-time way to monitor underreporting and an indication of the actual prevalence of an infectious illness in previous situations [34,35]. Nonetheless, mortality statistics may also be underreported in areas with insufficient or low testing levels, making CFR a less representative indication. Therefore, in this study, the simpler and more comprehensive factor *f*_2_ was utilized to estimate *Inf* (Eq2), based on test positivity rate (TPR) - an indication of appropriate testing in relation to disease prevalence (Eq3). The data on the number of tests per million was collected from WDI [37].

$$f_{2}=\left\{ \begin{aligned} 1 , if &TPR<0.05 \\ \frac{TPR}{0.05} , if &TPR\geq0.05 \end{aligned} \right.$$

(2)

$$TPR=\frac{C}{T}$$

(3)

When TPR is less than 5%, the World Health Organization (WHO) recommends that testing capacity is acceptable [40]. If TPR was more than 5 percent, it was assumed that a growing number of instances were being unreported and that the factor *f*_2_ would be equal to the TPR's ratio to that of 5 percent, the threshold (Eq2). The estimated *Inf* was used as a response variable in linear regression models in this study.

**Reference**

33. Kuster AC, Overgaard HJ. A novel comprehensive metric to assess effectiveness of COVID-19 testing: Inter-country comparison and association with geography, government, and policy response. PLoS One. 2021;16(3):e0248176. Epub 20210305. doi: 10.1371/journal.pone.0248176. PubMed PMID: 33667280; PubMed Central PMCID: PMCPMC7935311.

34. Fraser C, Donnelly CA, Cauchemez S, Hanage WP, Van Kerkhove MD, Hollingsworth TD, et al. Pandemic potential of a strain of influenza A (H1N1): early findings. Science. 2009;324(5934):1557-61. Epub 20090511. doi: 10.1126/science.1176062. PubMed PMID: 19433588; PubMed Central PMCID: PMCPMC3735127.

35. Nishiura H, Klinkenberg D, Roberts M, Heesterbeek JA. Early epidemiological assessment of the virulence of emerging infectious diseases: a case study of an influenza pandemic. PLoS One. 2009;4(8):e6852. Epub 20090831. doi: 10.1371/journal.pone.0006852. PubMed PMID: 19718434; PubMed Central PMCID: PMCPMC2729920.

37. World Development Indicators. Washington, USA: The World Bank; [cited 2021 October 1]. Available from: <https://databank.worldbank.org/home>.

40. Stein R, Wroth C, Hurt A. U.S. Coronavirus Testing Still Falls Short. How’s Your State Doing?2020 [cited 2022 01 July ]. Available from: <https://www.npr.org/sections/health-shots/2020/05/07/851610771/u-s-coronavirus-testing-still-falls-short-hows-your-state-doing>

**Before revision in ABSTRACT (line 30)**

A country-level ecological study using publicly available data of countries was conducted. We employed linear regression analysis to assess the association between COVID-19 and parasitic diseases. We considered demographic, socioeconomic, and geographic confounders previously suggested. A notable heterogeneity was observed across WHO regions. The countries in Africa (AFRO) showed the lowest rates of COVID-19 incidence, and the countries in the Americas (AMRO) presented the highest. The multivariable model results were computed using 165 countries, excluding missing values. In the models analyzed, lower COVID-19 incidence rates were consistently observed in malaria-endemic countries, even accounting for potential confounding variables (GDP per capita, population aged 65 and above, differences in the duration of COVID-19). However, the other parasitic diseases were not significantly associated with the spread of the pandemic.

**After revision in ABSTRACT (lines 30-31)**

A country-level ecological study using publicly available data of countries was conducted. **We conceptualized the true number of COVID-19 infections based on a function of test positivity rate (TPR) and employed linear regression analysis to assess the association between the outcome and parasitic diseases**. We considered demographic, socioeconomic, and geographic confounders previously suggested. A notable heterogeneity was observed across WHO regions. The countries in Africa (AFRO) showed the lowest rates of COVID-19 incidence, and the countries in the Americas (AMRO) presented the highest. The multivariable model results were computed using 165 countries, excluding missing values. In the models analyzed, lower COVID-19 incidence rates were consistently observed in malaria-endemic countries, even accounting for potential confounding variables, Gross Domestic Product (GDP) per capita, the population aged 65 and above, and differences in the duration of COVID-19. However, the other parasitic diseases were not significantly associated with the spread of the pandemic.

**Before revision in RESULTS (line 230)**

A multivariable model with parasitic diseases including malaria, schistosomiasis, and STH was analyzed as a fundamental model (Table 4). COVID-19 incidence rates were significantly lower in countries endemic for malaria (β coefficient = -0.86; 95 % CI of -1.44 to -0.44; *p* < 0.01) and STH (β coefficient = -0.73; 95 % CI of -1.12 to -0.36; *p* < 0.01). The countries where schistosomiasis is prevailing, on the other hand, were not revealed to have a significant association with COVID-19 incidence rates, though the coefficient was negative (β coefficient = -0.48; 95 % CI of -1.04 to 0.07; *p* = 0.07).

**After revision in RESULTS (lines 263-271)**

A multivariable model with parasitic diseases including malaria, schistosomiasis, and STHs was analyzed as a fundamental model (Table 4). COVID-19 incidence rates were significantly lower in countries endemic for malaria ~~(β coefficient = -0.86; 95 % CI of -1.44 to -0.44;~~ *~~p~~* ~~< 0.01)~~ **(β coefficient = -1.38; 95 % CI of -2.33 to -0.41; *p* < 0.01)** ~~and STH (β coefficient = -0.73; 95 % CI of -1.12 to -0.36;~~ *~~p~~* ~~< 0.01)~~. The countries where schistosomiasis or STHs are prevailing, on the other hand, were not revealed to have a significant association with COVID-19 incidence rates, though the coefficients were both negative for schistosomiasis ~~(β coefficient = -0.48; 95 % CI of -1.04 to 0.07;~~ *~~p~~* ~~= 0.07)~~ **(β coefficient = -0.84; 95 % CI of -1.76 to 0.15; *p* = 0.09) and STHs (β coefficient = -0.58; 95 % CI of -1.20 to 0.01; *p* = 0.05)**.

**Before revision in RESULTS (line 259)**

After screening significant variables using univariable analysis, variables with pairwise correlation coefficients between -0.7 and 0.7 were comprised for further multivariable linear regression (S1 Fig). The saturated model had the least value of AIC and BIC (S1 Table). Since the AIC and BIC significantly increased when the parasitic diseases variables were removed from the model, it could be concluded that the variables explain a considerable level of variation in the saturated model. A total of 165 countries were retained in the model, excluding missing values. Table 5 summarizes key results from the multivariable regression model of imputed predictive variables for the response variable, COVID-19 incidence rates. In the model, the malaria-endemic countries presented significantly lower COVID-19 incidence rates (β coefficient = -0.40; 95 % CI of -0.79 to -0.06; *p* < 0.05) even adjusting for confounding variables, the timing of the disease eruption (duration variable), the proportion of the population over 65, and GDP per capita, while schistosomiasis (β coefficient = -0.19; 95 % CI of -0.58 to 0.18; *p* = 0.31) and STH (β coefficient = -0.18; 95 % CI of -0.46 to 0.10; *p* = 0.21) were not significantly associated with the response variable. In addition, the model examined GDP per capita and the timing of the disease eruption to have significant associations for COVID-19 incidence rates. In the univariable regression model, including a predictor, the prevalence of malaria, and an outcome, COVID-19 incidence rates, the outcome variable monotonically decreased as the predictor increased (β coefficient = -0.02; 95 % CI of -0.04 to -0.01; *p* < 0.01) (Fig 3).

**After revision in RESULTS (lines 292-310)**

After screening significant variables using univariable analysis, variables with pairwise correlation coefficients between -0.7 and 0.7 were comprised for further multivariable linear regression (S1 Fig). The saturated model had the least value of AIC and BIC (S1 Table). Since the AIC and BIC significantly increased when the parasitic disease variables were removed from the model, it could be concluded that the variables explain a considerable level of variation in the saturated model. A total of 165 countries were retained in the model, excluding missing values. Table 5 summarizes key results from the multivariable regression model of imputed predictive variables for the response variable, COVID-19 incidence rates. In the model, the malaria-endemic countries presented significantly lower COVID-19 incidence rates ~~(β coefficient = -0.40; 95 % CI of -0.79 to -0.06;~~ *~~p~~* ~~< 0.05)~~ **(β coefficient = -0.73; 95 % CI of -1.46 to -0.10; *p* < 0.05)** even adjusting for confounding variables, the timing of the disease eruption (duration variable), the proportion of the population over 65, and GDP per capita, while schistosomiasis ~~(β coefficient = -0.19; 95 % CI of -0.58 to 0.18;~~ *~~p~~* ~~= 0.31)~~ **(β coefficient = -0.30; 95 % CI of -1.02 to 0.40; *p* = 0.39) and STHs** ~~(β coefficient = -0.18; 95 % CI of -0.46 to 0.10;~~ *~~p~~* ~~= 0.21)~~ **(β coefficient = -0.14; 95 % CI of -0.67 to 0.39; *p* = 0.59)** were not significantly associated with the response variable. In addition, the model examined GDP per capita ~~and the timing of the disease eruption~~ to have significant associations for COVID-19 incidence rates. In the univariable regression model ~~including a predictor,~~ with the prevalence of malaria as a predictor, and COVID-19 incidence rates as an outcome, the outcome variable monotonically decreased as the predictor increased **(β coefficient = -0.02; 95 % CI of -0.04 to -0.01; *p* < 0.01)** (Fig 3).

**Before revision in DISCUSSION (line 398)**

Due to the differences in health infrastructure and testing policy, the data may obscure the accurate size of the diseases [5]. Studies have revealed significantly under-reported cases and associated deaths in several countries, especially in low-income countries [55]. Thus, our results should be cautiously interpreted. We also want to point out that we might not have adjusted for all confounding variables, though we attempted to include demographic, socioeconomic, and geographic variables suggested in previous studies. Our study is based on an analysis describing a still evolving and imperfectly explored epidemic, only considering the scientific understanding of the disease at the time. Therefore, due to these unknown factors, our study could not deal with all factors that influence the spread of COVID-19 and residual confounding.

**After revision in DISCUSSION (lines 435-445)**

Due to the differences in health infrastructure and testing policy, the data may obscure the accurate size of the diseases [5]. Studies have revealed significantly under-reported cases and associated deaths in several countries, especially in low-income countries [65]. **Even though we conceptualized the true number of the infections based on TPR to adjust possible biases in the under-reporting of the COVID-19 cases, we cannot conclude that our study has perfectly precluded them.** Thus, our results should be cautiously interpreted. We also want to point out that we might not have adjusted for all confounding variables, though we attempted to include demographic, socioeconomic, and geographic variables suggested in previous studies. Our study is based on an analysis describing a still evolving and imperfectly explored epidemic, only considering the scientific understanding of the disease at the time. Therefore, due to these unknown factors, our study could not deal with all factors that influence the spread of COVID-19 and residual confounding.

**Table 3. Univariable analyses of predictive variables and root-transformed COVID-19 incidence rates.**

| **Variable** | **Number of countries examined (%)^1^** | **Coefficient (95% CI)** | **SE of coefficient** | **Coefficient (95% CI) /bootstrapping** | **SE of coefficient /bootstrapping** | ***p*-value of coefficient^2^** | **ΔR^2^** |
| --- | --- | --- | --- | --- | --- | --- | --- |

| **Malaria (in %)** |  | |  | |  | |  | |  | |  | |  | |  |  |
| --- | --- | --- | --- | --- | --- | --- | --- | --- | --- | --- | --- | --- | --- | --- | --- | --- |
| Non-endemic | 156 (78.79) | | Reference | |  | | Reference | |  | |  | |  | | | |
| Endemic | 42 (21.21) | | ~~-1.59 (-1.96, -1.23)~~  -2.29 (-2.89, -1.69) | | ~~0.18~~  0.30 | | ~~-1.59 (-1.82, -1.36)~~  -2.29 (-2.64, -1.86) | | ~~0.12~~  0.19 | | ~~< 0.001~~  < 0.001 | | ~~0.28~~  0.23 | | | |
| **Schistosomiasis (in %)** |  | |  | |  | |  | |  | |  | |  | | | |
| Non-endemic | 157 (77.73) | | Reference | |  | | Reference | |  | |  | |  | | | |
| Endemic | 45 (22.27) | | ~~-1.53 (-1.89, -1.17)~~  -2.19 (-2.82, -1.56) | | ~~0.18~~  0.31 | | ~~-1.53 (-1.75, -1.24)~~  -2.19 (-2.63, -1.75) | | ~~0.13~~  0.30 | | ~~< 0.001~~  < 0.001 | | ~~0.26~~  0.19 | | | |
| **STHs (in %)** |  | |  | |  | |  | |  | |  | |  | | | |
| Non-endemic | 146 (73.74) | | Reference | |  | | Reference | |  | |  | |  | | | |
| Endemic | 52 (26.26) | | ~~-1.28 (-1.63, -0.93)~~  -1.49 (-2.13, -0.85) | | ~~0.18~~  0.32 | | ~~-1.28 (-1.56, -0.98)~~  -1.49 (-2.06, -0.86) | | ~~0.14~~  0.30 | | ~~< 0.001~~  < 0.001 | | ~~0.2~~  0.11 | | | |
| **Tuberculosis (in %)** |  | |  | |  | |  | |  | |  | |  | | | |
| Low (< 0.01) | 55 (29.41) | | Reference | |  | | Reference | |  | |  | |  | | | |
| Moderate (< 0.1) | 91 (48.66) | | ~~-0.97 (-1.33, -0.61)~~  -0.99 (-1.63, -0.35) | | ~~0.18~~  0.32 | | ~~-0.97 (-1.33, -0.61)~~  -0.99 (-1.77, -0.61) | | ~~0.19~~  0.36 | | ~~< 0.001~~  < 0.001 | | ~~0.27~~  0.17 | | | |
| High (0.1 ≤) | 41 (21.93) | | ~~-1.82 (-2.26, -1.39)~~  -2.38 (-3.16, -1.61) | | ~~0.22~~  0.39 | | ~~-1.82 (-2.19, -1.46)~~  -2.38 (-3.11, -1.72) | | ~~0.19~~  0.34 | | ~~< 0.001~~  < 0.001 | |  | | | |
| **Population ages 65 and above (in %)** |  | |  | |  | |  | |  | |  | |  | | | |
| < 5 | 72 (38.30) | | Reference | |  | | Reference | |  | |  | |  | | | |
| < 15 | 70 (37.23) | | ~~1.13 (0.81, 1.45)~~  2.15 (1.54, 2.75) | | ~~0.16~~  0.35 | | ~~1.13 (0.77, 1.46)~~  2.15 (1.54, 2.80) | | ~~0.16~~  0.34 | | ~~< 0.001~~  < 0.001 | | ~~0.37~~  0.26 | | | |
| 15 ≤ | 46 (24.47) | | ~~1.87 (1.51, 2.24)~~  2.28 (1.60, 2.97) | | ~~0.18~~  0.32 | | ~~1.87 (1.51, 2.19)~~  2.28 (1.74, 2.88) | | ~~0.16~~  0.28 | | ~~< 0.001~~  < 0.001 | |  | | | |
| **Death by non-communicable diseases (in %)** | |  | |  | |  | |  | |  | |  | |  | | |
| < 60 | | 50 (27.17) | | Reference | |  | | Reference | |  | |  | |  | | |
| < 80 | | 54 (29.35) | | ~~1.04 (0.67, 1.41)~~  1.96 (1.26, 2.65) | | ~~0.18~~  0.35 | | ~~1.04 (0.71, 1.38)~~  1.96 (1.32, 2.70) | | ~~0.17~~  0.17 | | ~~< 0.001~~  < 0.001 | | ~~0.41~~  0.25 | | |
| 80 ≤ | | 80 (43.48) | | ~~1.92 (1.57, 2.25)~~  2.51 (1.87, 3.15) | | ~~0.17~~  0.32 | | ~~1.92 (1.61, 2.18)~~  2.51 (2.03, 2.96) | | ~~0.14~~  0.14 | | ~~< 0.001~~  < 0.001 | |  | | |
| **Diabetes (in %)** | |  | |  | |  | |  | |  | |  | |  | |  |
| < 5 | | 39 (19.31) | | Reference | |  | | Reference | |  | |  | |  | |  |
| < 10 | | 103 (50.99) | | ~~0.68 (0.23, 1.14)~~  1.17 (0.41, 1.93) | | ~~0.23~~  0.38 | | ~~0.68 (0.18, 1.02)~~  1.17 (0.53, 1.78) | | ~~0.23~~  0.31 | | ~~< 0.01~~  < 0.01 | | ~~0.04~~  0.05 | |  |
| 10 ≤ | | 60 (29.70) | | ~~0.55 (0.05, 1.04)~~  1.13 (0.29, 1.96) | | ~~0.25~~  0.41 | | ~~0.55 (0.05, 1.02)~~  1.13 (0.38, 1.98) | | ~~0.25~~  0.40 | | ~~0.03~~  < 0.01 | |  | |  |
| **GDP per capita (in $)** | |  | |  | |  | |  | |  | |  | |  | | |
| < 2000 | | 48 (24.87) | | Reference | |  | | Reference | |  | |  | |  | | |
| < 6,000 | | 43 (22.28) | | ~~0.96 (0.58, 1.35)~~  1.70 (0.99, 2.43) | | ~~0.19~~  0.36 | | ~~0.96 (0.63, 1.33)~~  1.70 (1.13, 2.36) | | ~~0.16~~  0.30 | | ~~< 0.001~~  < 0.001 | | ~~0.44~~  0.31 | | |
| < 18,000 | | 53 (27.46) | | ~~1.83 (1.46, 2.19)~~  3.02 (2.33, 3.71) | | ~~0.19~~  0.34 | | ~~1.83 (1.54, 2.14)~~  3.02 (2.41, 3.73) | | ~~0.15~~  0.33 | | ~~< 0.001~~  < 0.001 | |  | |  |
| 18,000 ≤ | | 49 (25.39) | | ~~2.13 (1.76, 2.50)~~  2.26 (1.56, 2.96) | | ~~0.19~~  0.35 | | ~~2.13 (1.83, 2.43)~~  2.26 (1.86, 2.69) | | ~~0.15~~  0.21 | | ~~< 0.001~~  < 0.001 | |  | |  |
| **Current health expenditure (% GDP)** | |  | |  | |  | |  | |  | |  | |  | |  |
| < 5 | | 60 (38.08) | | Reference | |  | | Reference | |  | |  | |  | |  |
| < 8 | | 81 (43.32) | | ~~0.84 (0.45, 1.24)~~  1.59 (0.94, 2.24) | | ~~0.19~~  0.32 | | ~~0.84 (0.47, 1.21)~~  1.59 (0.94, 2.24) | | ~~0.19~~  0.27 | | ~~< 0.001~~  < 0.001 | | ~~0.13~~  0.14 | |  |
| 8 ≤ | | 46 (24.60) | | ~~1.11 (0.65, 1.55)~~  1.74 (1.01, 2.49) | | ~~0.23~~  0.37 | | ~~1.11 (0.64, 1.53)~~  1.74 (1.01, 2.49) | | ~~0.22~~  0.35 | | ~~< 0.001~~  < 0.001 | |  | |  |
| **Average surface temperature (in ℃)** | |  | |  | |  | |  | |  | |  | |  | |  |
| Polar-Microthermal (< 18) | | 66 (33.67) | | Reference | |  | | Reference | |  | |  | |  | |  |
| Temperate (< 26) | | 60 (30.61) | | ~~-1.25 (-1.64, -0.86)~~  -1.25 (-1.93, -0.51) | | ~~0.20~~  0.36 | | ~~-1.25 (-1.61, -0.89)~~  -1.25 (-1.85, -0.55) | | ~~0.18~~  0.33 | | ~~< 0.001~~  < 0.001 | | ~~0.2~~  0.11 | |  |
| Tropical (≤ 26) | | 70 (35.71) | | ~~-1.10 (-1.48, -0.73)~~  -1.27 (-1.96, -0.58) | | ~~0.19~~  0.34 | | ~~-1.10 (-1.44, -0.68)~~  -1.27 (-1.88, -0.44) | | ~~0.20~~  0.36 | | ~~< 0.001~~  < 0.001 | |  | |  |
| **Urban population (in %)** | |  | |  | |  | |  | |  | |  | |  | |  |
| < 50 | | 67 (33.17) | | Reference | |  | | Reference | |  | |  | |  | |  |
| < 75 | | 72 (35.64) | | ~~1.03 (0.66, 1.41)~~  1.91 (1.27, 2.56) | | ~~0.19~~  0.32 | | ~~1.03 (0.63, 1.67)~~  1.91 (1.33, 2.60) | | ~~0.19~~  0.32 | | ~~< 0.001~~  < 0.001 | | ~~0.21~~  0.17 | |  |
| 75 ≤ | | 63 (31.19) | | ~~1.33 (0.94, 1.72)~~  1.69 (1.02, 2.35) | | ~~0.20~~  0.33 | | ~~1.33 (0.96, 1.67)~~  1.69 (1.15, 2.24) | | ~~0.18~~  0.27 | | ~~< 0.001~~  < 0.001 | |  | |  |
| **Fully vaccinated with COVID-vaccine (in %)** | |  | |  | |  | |  | |  | |  | |  | |  |
| < 30 | | 86 (43.21) | | Reference | |  | | Reference | |  | |  | |  | |  |
| < 60 | | 61 (30.65) | | ~~1.36 (1.02, 1.71)~~  2.20 (1.58, 2.82) | | ~~0.17~~  0.31 | | ~~1.36 (1.02, 1.69)~~  2.20 (1.55, 2.82) | | ~~0.17~~  0.32 | | ~~< 0.001~~  < 0.001 | | ~~0.31~~  0.30 | |  |
| 60 ≤ | | 52 (26.13) | | ~~1.42 (1.06, 1.79)~~  1.27 (0.67, 1.92) | | ~~0.18~~  0.33 | | ~~1.42 (1.05, 1.80)~~  1.27 (0.71, 2.11) | | ~~0.19~~  0.33 | | ~~< 0.001~~  < 0.001 | |  | |  |
| **Population density (per km²)** | |  | |  | |  | |  | |  | |  | |  | |  |
| < 50 | | 67 (33.83) | | Reference | |  | | Reference | |  | |  | |  | |  |
| < 120 | | 59 (29.80) | | ~~0.21 (-0.23, 0.65)~~  0.36 (-0.37, 1.65) | | ~~0.22~~  0.37 | | ~~0.21 (-0.23, 0.63)~~  0.36 (-0.36, 1.45) | | ~~0.22~~  0.34 | | ~~0.34~~  0.34 | | ~~0.02~~  0.02 | |  |
| 120 ≤ | | 72 (36.36) | | ~~0.37 (-0.05, 0.78)~~  0.20 (-0.50, 0.98) | | ~~0.21~~  0.35 | | ~~0.37 (-0.03, 0.78)~~  0.20 (-0.47, 0.88) | | ~~0.21~~  0.36 | | ~~0.08~~  0.57 | |  | |  |
| **Human development index** | |  | |  | |  | |  | |  | |  | |  | |  |
| < 0.6 | | 46 (24.60) | | Reference | |  | | Reference | |  | |  | |  | |  |
| < 0.8 | | 76 (40.64) | | ~~1.32 (1.01, 1.64)~~  2.43 (1.80, 3.06) | | ~~0.16~~  0.32 | | ~~1.32 (1.08, 1.56)~~  2.43 (1.93, 2.97) | | ~~0.13~~  0.32 | | ~~< 0.001~~  < 0.001 | | ~~0.52~~  0.31 | |  |
| 0.8 ≤ | | 65 (34.76) | | ~~2.33 (2.01, 2.66)~~  2.85 (2.20, 3.51) | | ~~0.17~~  0.33 | | ~~2.33 (2.07, 2.57)~~  2.85 (2.48, 3.33) | | ~~0.12~~  0.33 | | ~~< 0.001~~  < 0.001 | |  | |  |
| **Duration (in days)** | |  | |  | |  | |  | |  | |  | |  | |  |
| < 70 | | 104 (52.26) | | Reference | |  | | Reference | |  | |  | |  | |  |
| 70 ≤ | | 95 (47.74) | | ~~-1.10 (-1.41, -0.79)~~  -1.34 (-1.89, -0.79) | | ~~0.16~~  0.28 | | ~~-1.10 (-1.39, -0.77)~~  -1.34 (-1.89, -0.78) | | ~~0.15~~  0.27 | | ~~< 0.001~~  < 0.001 | | ~~0.19~~  0.19 | |  |
| **Mean years of schooling (in years)** | |  | |  | |  | |  | |  | |  | |  | |  |
| < 7 | | 58 (31.35) | | Reference | |  | | Reference | |  | |  | |  | |  |
| < 10 | | 50 (27.03) | | ~~1.40 (1.04, 1.77)~~  1.59 (1.04, 1.77) | | ~~0.18~~  0.33 | | ~~1.40 (1.09, 1.71)~~  1.59 (1.11, 1.67) | | ~~0.13~~  0.28 | | ~~< 0.001~~  < 0.001 | | ~~0.41~~  0.30 | |  |
| 10 ≤ | | 77 (41.62) | | ~~1.87 (1.54, 2.20)~~  1.97 (1.54, 2.20) | | ~~0.17~~  0.29 | | ~~1.87 (1.58, 2.15)~~  1.97 (1.64, 2.19) | | ~~0.12~~  0.31 | | ~~< 0.001~~  < 0.001 | |  | |  |

^1^Percentages reflect cases without missing values

^2^The level of statistical significance is 0.05

**Table 4. Summary of associations between parasitic disease variables and root-transformed COVID-19 incidence rates. (changed)**

| **Variable^1^** | | **Coefficient (95% CI)** | | **SE of coefficient** | | **Coefficient (95% CI) /bootstrapping** | | **SE of coefficient /bootstrapping** | | ***p-*value of coefficient** | | **Adjusted ΔR^2^** | | ***p-*value of ΔR^2^** |
| --- | --- | --- | --- | --- | --- | --- | --- | --- | --- | --- | --- | --- | --- | --- |
| **Malaria (in %)** |  | |  | |  | |  | |  | |  | |  | |
| Non-endemic | Reference | |  | | Reference | |  | |  | |  | |  | |
| Endemic | ~~-0.86 (-1.44, -0.44)~~  -1.38 (-2.33, -0.41) | | ~~0.28~~  0.48 | | ~~-0.86 (-1.37, -0.40)~~  -1.38 (-2.06, -0.63) | | ~~0.25~~  0.36 | | ~~< 0.01~~  < 0.01 | |  | |  | |
| **Schistosomiasis (in %)** |  | |  | |  | |  | |  | |  | |  | |
| Non-endemic | Reference | |  | | Reference | |  | |  | | 0.24 | | < 0.001 | |
| Endemic | ~~-0.48 (-1.04, 0.07)~~  -0.84 (-1.76, 0.15) | | ~~0.29~~  0.48 | | ~~-0.48 (-0.98, 0.00)~~  -0.84 (-1.57, -0.04) | | ~~0.26~~  0.38 | | ~~0.07~~  0.09 | |  | |  | |
| **STHs (in %)** |  | |  | |  | |  | |  | |  | |  | |
| Non-endemic | Reference | |  | | Reference | |  | |  | |  | |  | |
| Endemic | ~~-0.73 (-1.12, -0.36)~~  -0.58 (-1.20, 0.01) | | ~~0.19~~  0.32 | | ~~-0.73 (-1.04, -0.45)~~  -0.58 (-1.15, 0.02) | | ~~0.16~~  0.32 | | ~~< 0.01~~  0.05 | |  | |  | |

^1^Interaction terms were not significant

**Table 5.** **Summary of multivariable linear regression, including parasite variables of interest and potential confounders. (changed)**

| **Variable** | | **Coefficient (95% CI)** | | **SE of coefficient** | | **Coefficient (95% CI) /bootstrapping** | **SE of coefficient /bootstrapping** | | ***p-*value of coefficient** | | **Adjusted ΔR^2^** | | ***p-*value of ΔR^2^** | |
| --- | --- | --- | --- | --- | --- | --- | --- | --- | --- | --- | --- | --- | --- | --- |
| **Malaria** |  | |  | |  | | |  | |  | |  | |  |
| Non-endemic | Reference | |  | | Reference | | |  | |  | |  | |  |
| Endemic | ~~-0.40 (-0.79, -0.06)~~  -0.73 (-1.46, -0.10) | | ~~0.2~~  0.36 | | ~~-0.40 (-0.83, -0.12)~~  -0.73 (-1.42, -0.12) | | | ~~0.18~~  0.34 | | ~~< 0.05~~  < 0.05 | |  | |  |
| **Schistosomiasis** |  | |  | |  | | |  | |  | |  | |  |
| Non-endemic | Reference | |  | | Reference | | |  | |  | |  | |  |
| Endemic | ~~-0.19 (-0.58, 0.18)~~  -0.30 (-1.02, 0.40) | | ~~0.2~~  0.36 | | ~~-0.19 (-0.48, 0.05)~~  -0.30 (-1.06, 0.34) | | | ~~0.19~~  0.36 | | ~~0.31~~  0.39 | |  | |  |
| **STHs** |  | |  | |  | | |  | |  | |  | |  |
| Non-endemic | Reference | |  | | Reference | | |  | |  | |  | |  |
| Endemic | ~~-0.18 (-0.46, 0.10)~~  -0.14 (-0.67, 0.39) | | ~~0.15~~  0.26 | | ~~-0.18 (-0.48, 0.01)~~  -0.14 (-0.64, 0.45) | | | ~~0.12~~  0.28 | | ~~0.21~~  0.59 | |  | |  |
| **Duration (in days)** |  | |  | |  | | |  | |  | |  | |  |
| < 70 | Reference | |  | | Reference | | |  | |  | | ~~0.69~~  0.49 | | ~~< 0.001~~  < 0.001 |
| 70 ≤ | ~~-0.36 (-0.61, -0.10)~~  -0.40 (-0.87, 0.07) | | ~~0.12~~  0.24 | | -~~0.36 (-0.41, -0.12)~~  -0.40 (-0.88, 0.09) | | | ~~0.11~~  0.29 | | ~~< 0.01~~  0.11 | |  | |  |
| **Population ages 65 and above (in %)** |  | |  | |  | | |  | |  | |  | |  |
| < 5 | Reference | |  | | Reference | | |  | |  | |  | |  |
| < 15 | ~~-0.06 (-0.34, 0.28)~~  0.17(-0.41, 0.74) | | ~~0.16~~  0.29 | | ~~-0.06 (-0.40, 0.35)~~  0.17(-0.45, 0.80) | | | ~~0.19~~  0.33 | | ~~0.86~~  0.56 | |  | |  |
| 15 ≤ | ~~0.23 (-0.13, 0.63)~~  0.35 (-0.38, 1.04) | | ~~0.19~~  0.35 | | ~~0.23 (-0.17, 0.72)~~  0.35 (-0.35, 1.02) | | | ~~0.22~~  0.36 | | ~~0.20~~  0.36 | |  | |  |
| **GDP per capita (in $)** |  | |  | |  | | |  | |  | |  | |  |
| < 2000 | Reference | |  | | Reference | | |  | |  | |  | |  |
| < 6,000 | ~~0.43 (0.10, 0.77)~~  0.88 (0.25, 1.51) | | ~~0.15~~  0.31 | | ~~0.43 (0.15, 0.69)~~  0.88 (0.26, 1.58) | | | ~~0.15~~  0.32 | | ~~< 0.05~~  < 0.001 | |  | |  |
| < 18,000 | ~~1.3 (0.94, 1.72)~~  1.86 (1.12, 2.59) | | ~~0.18~~  0.37 | | ~~1.3 (0.94, 1.61)~~  1.86 (1.26, 2.64) | | | ~~0.21~~  0.34 | | ~~< 0.001~~  < 0.001 | |  | |  |
| 18,000 ≤ | ~~0.95 (0.95, 1.87)~~  0.96 (0.12, 1.80) | | ~~0.19~~  0.41 | | ~~0.95 (0.98, 1.52)~~  0.96 (0.22, 1.78) | | | ~~0.22~~  0.39 | | ~~< 0.001~~  < 0.05 | |  | |  |
